# Structured multimodal deep learning improves genomic prediction in future environments

**DOI:** 10.1093/genetics/iyag204

**Published:** 2026-08-11

**Authors:** Aike Potze, Fred van Eeuwijk, Ioannis N Athanasiadis

**Affiliations:** Artificial Intelligence Group, Wageningen University and Research, Wageningen, 6708 PB, The Netherlands; Mathematical and Statistical Methods Group (Biometris), Wageningen University and Research, Wageningen, 6708 PB, The Netherlands; Artificial Intelligence Group, Wageningen University and Research, Wageningen, 6708 PB, The Netherlands

**Keywords:** genomic selection, GxExM, multimodal deep learning, genomic prediction, genotype-by-environment interaction, plant breeding, Plantae

## Abstract

Predicting phenotypes from genetic and environmental information is a long-standing challenge in genetics and plant breeding. Deep neural networks are a promising approach due to their capacity to approximate nonlinear biological processes. Despite initial expectations, recent studies have found that deep neural networks under-perform compared to linear methods, even on continent-scale datasets. We attribute this to several failure modes of deep learning, including *greedy learning*, the tendency to over-emphasize a single type of input data. As a solution, we present the structured interaction neural network (SINN), which combines statistical decomposition of genetic, environmental and interaction effects with deep neural networks. SINN dissects phenotype prediction into isolated component modeling tasks, revealing poor generalization of learned representations to new environments as the main limitation for prediction of genotype-by-environment interactions and overall yield. We reach competitive performance on yield prediction in the next cycle of 2 maize multi-environment trial datasets, including new genotypes and environments. In the Genomes to Fields (United States) dataset, SINN achieved higher overall accuracy (0.63) than BLUP-based methods (0.43) and a neural network from previous literature (0.48), and surpassed previous top-performing models with a lower RMSE (2.40 vs. 2.46 Mg/ha, mean yield 9.51 Mg/ha). Similar gains were observed in a secondary dataset consisting of Chinese national maize variety trials (MaizeGEP). SINN achieved higher overall accuracy (0.79) than BLUP-based methods (0.49) and a neural network from previous literature (0.76). By combining statistical genetics and modern deep learning, SINN enables accurate, modular and scalable genomic prediction in new environments.

## Introduction

Climate change is shifting local environmental conditions, which increasingly threatens global crop yields (Kang et al. 2009). A major focus of modern plant breeding is the mitigation of these effects of climate change on crop performance (Chapman et al. 2012). Climate change can also directly affect the effectiveness of breeding programs, as it can reduce alignment between a multi-environment trial (MET) and the target population of environments (TPE) (Cooper et al. 2023). To address this challenge, new methods are needed that predict how new crop genotypes and environments lead to a desired phenotype. As this task forms an extension of genomic prediction (GP) (Meuwissen et al. 2001) to new environments, we refer to this prediction task as genomic–enviromic prediction (GEP).

Interest in GEP has surged in recent years, inspiring a diverse set of novel methods. Proposed statistical methods include factorial regression on environmental covariates (ECs) (Heslot et al. 2014), linear mixed models using enviromic relationship kernels (Jarquín et al. 2014), linear and linear mixed models using grouping of environments in envirotypes (Xu 2016) and mega-scale linear mixed models (Hu et al. 2025). Additionally, various methods incorporating process-based crop growth models (Jullien et al. 2011; Technow et al. 2015; Cooper et al. 2016; Rincent et al. 2019) and machine learning models (Westhues et al. 2021; Fernandes et al. 2024; He et al. 2025) have been proposed.

Recently, multimodal deep learning (MMDL), a category of deep learning methods combining multiple types (modalities) of data, has emerged as a promising paradigm for genomic prediction (Montesinos-López et al. 2024) due to its capacity to model nonlinear relationships (Goodfellow et al. 2016), improvements in predictive performance with dataset size (Hestness et al. 2017), and flexibility in combining diverse data (Ngiam et al. 2011). This flexibility enables integration of diverse omics data into genomic prediction, with examples including diverse phenomics (Montesinos-López et al. 2023; Togninalli et al. 2023), environmics (Montesinos-López et al. 2018; Khaki and Wang 2019), transcriptomics, epigenomics and proteomics (Zhao et al. 2022).

Adoption of MMDL for GEP has been relatively limited (Jubair and Domaratzki 2023), with mixed success in case studies for wheat (Montesinos-López et al. 2019; J Guo et al. 2020; Sandhu et al. 2021; Jubair et al. 2023), maize (Montesinos-López et al. 2018; Khaki and Wang 2019; Washburn et al. 2021; Kick et al. 2023; Yao et al. 2025; D Zhang et al. 2026) and barley (Måløy et al. 2021). Despite the widespread optimism and the isolated improvements reported in aforementioned works, open benchmarking of models for GEP on a continent-scale MET found MMDL methods not to be competitive with machine learning and statistical methods (Washburn et al. 2025). Considering the massive scale of this data, with over 100,000 measured plots (Lima et al. 2023), this suggests that current MMDL approaches are ineffective at GEP for even the largest of current METs.

Current MMDL research tempers the aforementioned optimism: multimodal models do not necessarily improve over unimodal approaches, and are not guaranteed to learn any interactions. Multimodal architectures are more complex than unimodal architectures, which harms performance through *overfitting* (W Wang et al. 2020). The rate of this overfitting is modality-specific, which can contribute to *greedy learning*, the tendency to over-rely on a single dominant modality while underfitting on additional modalities (Cadene et al. 2019; Huang et al. 2022; Peng et al. 2022; Wu et al. 2022; Du et al. 2023; Makino et al. 2023; Y Zhang et al. 2024). Counterintuively, while multimodal models are often motivated from the prediction of interactions, they can function like the sum of unimodal models (Hessel and Lee 2020; Wörtwein et al. 2022), which indicates *underutilization of interactions*. Clearly, imbalanced multimodal learning introduces problem-specific failure mechanisms. Unfortunately, it remains an open question which specific failure mechanisms apply to GEP.

Both imbalanced multimodal learning (Wu et al. 2022) and the inconsistent performance of MMDL methods in GEP (Montesinos-López et al. 2024) are well-documented phenomena. We however argue that they are connected: imbalanced multimodal learning harms MMDL performance in GEP. We propose a new training algorithm specifically targeted at avoiding imbalanced multimodal learning in GEP, by leveraging the MET structure to guide neural network training. We name our training algorithm the *Structured Interaction Neural Network* (SINN). It contains 4 steps:

- First, we utilize the MET structure and statistical models to decompose phenotype into unimodal (genetic, environmental) and multimodal signal components (genotype-environment interactions).
- Second, the decomposed signals are used to train unimodal deep neural networks (DNNs) for genomic prediction of the genetic component and enviromic prediction of the environmental component.
- Third, the unimodal DNNs are used to initialize a multimodal DNN that estimates the genotype-environment interactions.
- Finally, the predictions of all 3 DNNs are summed to obtain predictions for new phenotypes.

By separating unimodal and multimodal signals, our SINN approach sidesteps greedy learning and underutilization of interactions. Furthermore, the structured optimization strategy enables us to finely tune the complexity of each component DNN to each modality, reducing overfitting.

We demonstrate the effectiveness of SINN by predicting the yield of new maize hybrids in the next year of 2 large-scale MET datasets; spanning both the United States and China. We first provide a detailed comparison of models using a U.S. benchmark dataset (Lima et al. 2023), collected and shared through the Genomes-to-Fields (G2F) Initiative (Lawrence-Dill et al. 2019). By following the benchmarking protocols of a 2022 competition with identical data (Washburn et al. 2025), we enable a fair comparison against 124 models submitted in the competition. To assess the generality of SINN across multiple breeding contexts and geographic settings, we further demonstrate its capacity on the MaizeGEP dataset (D Zhang et al. 2026) sourced from Chinese Regional Maize Variety Trials and following a protocol similar to the Genomes-to-Fields competition. It contains records of 260 hybrid maize varieties and 2,382 trials spanning 2017–2023. Jointly these experiments provide a comprehensive assessment of SINN’s performance, interpretability and generalization.

In short, we present SINN, a decomposition-based neural network framework for GEP of phenotype that:

- Improves prediction of yield for new maize genotypes in new environments
- Increases utilization of genetic information of DNNs
- Simplifies the development of multimodal DNNs
- Supports targeted improvement of genotype-environment interaction prediction

## Materials and methods

In this section, we describe our approach to yield prediction for new genotypes and new environments. Figure 1 shows an overview of the approach. Let $i=1,2,\ldots ,n_{g}$ denote the $n_{g}$ crop genotypes and $j=1,2,\ldots ,n_{e}$ denote the $n_{e}$ growing environments. For each genotype *i* we define a vector of $d_{g}$ genetic markers $x_{i}^{g}\in R^{d_{g}}$, representing genetic variations across the crop genome. For each environment *j* we define a vector of $d_{e}$ environmental covariates $x_{j}^{e}\in R^{d_{e}}$, representing observations of weather, soil and management variables. Furthermore, we define the crop yield of genotype *i* in environment *j* as $y_{ij}\in R_{\geq \;0}$. We then formalize the objective as follows. Given a set of $n_{s}$ training observations $S=\{(x_{s}^{g},x_{s}^{e},y_{s}){\}}_{s=1}^{n_{s}}$, learn a mapping *f* from genetic markers $x_{i}^{g}$ and environmental covariates $x_{j}^{e}$ to crop yield *y* for any any genotype-environment combination (*i*, *j*).

**Fig. 1.**
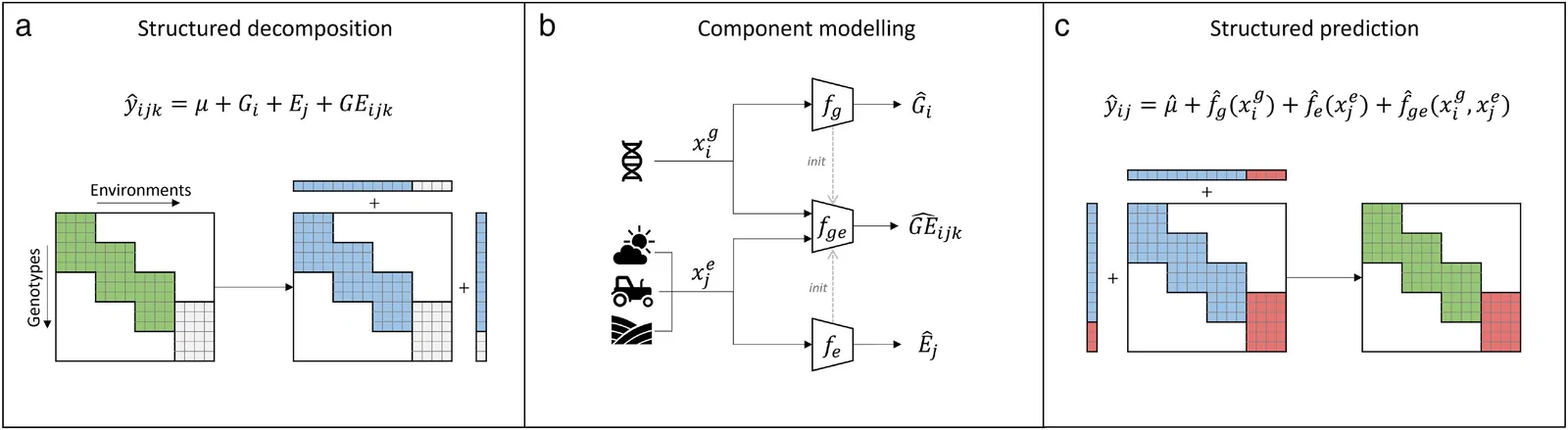
Overview of the proposed Structured Interaction Neural Network (SINN). a) Observed plot yields (green) are decomposed into yield components for genotypes, environments and interactions (blue). b) Estimated components are used to train 3 DNNs, which predict yield component from genetic markers ($x_{i}^{g}$) and environmental covariates ($x_{j}^{e}$), including weather, management and soil. The DNNs for genetic and environmental components are used to initialize the DNN for the interaction component, denoted by *init*. c) Trained DNNs predict each component for new genotypes and environments (red). Finally, DNN predictions are recomposed to predict the yield for unobserved plots in the next cycle of a multi-environment trial.

We define the quality of mapping *f* as the difference between predicted yield $f(x_{i}^{g},x_{j}^{e})$ and observed yield *y* on a set of held-out samples. We distinguish between 4 prediction or cross-validation (CV) scenarios, depending on whether genotype *i* and environment *j* were previously observed in S:

1. **CV-X**: Observed genotypes, observed environments
2. **CV-G**: New genotypes, observed environments
3. **CV-E**: Observed genotypes, new environments
4. **CV-GE**: New genotypes, new environments

While all 4 scenarios serve useful applications, they are not equally conductive to the comparison of flexible predictive models. Repeated structures, such as previously observed genotypes, can be exploited as shortcuts by flexible models, including neural networks. This leads to an overestimation of the true predictive ability when comparing models on scenario CV-X, CV-G and CV-E. As such, we utilized the most stringent scenario CV-GE to compare the ability of various models to generalize yield prediction to new conditions.

### Introducing SINN

Direct approximation of yield can lead to overfitting (W Wang et al. 2020), greedy learning (Wu et al. 2022) and underutilization of interactions (Hessel and Lee 2020). To avoid this issue, we do not directly approximate *y*, but instead define the following decomposed representation:


(1) $$y=\mu +f_{g}(x^{g})+f_{e}(x^{e})+f_{ge}(x^{g},x^{e}),$$

with components further defined as:


(2a ) μ=E[y],

(2b ) $$f_{g}(x^{g})=E[\;y|x^{g}\;]-\mu ,$$

(2c ) $$f_{e}(x^{e})=E[\;y|x^{e}\;]-\mu ,$$

(2d ) $$f_{ge}(x^{g},x^{e})=y-\mu -f_{g}(x^{g})-f_{e}(x^{e}),$$

where *μ* represents the overall expectation of *y*, the 2 component functions $f_{g}$ and $f_{e}$, represent the unimodal contributions of the genetic and environmental features, and $f_{ge}$ represents the contribution of genotype-by-environment interactions to *y*. Because genetic markers $x^{g}$ and environmental covariates $x^{e}$ are indexed by genotype *i* and environment *j*, respectively, we can approximate conditional expectation $E[\;y|x^{g}\;]$ by E[y∣i] and conditional expectation $E[\;y|x^{e}\;]$ by E[y∣j]. We subsequently estimate *μ*, E[y∣i] and E[y∣j] using the following linear fixed model:


(3) $$y_{ijk}=\mu +G_{i}+E_{j}+GE_{ijk},$$

where *μ* represents the grand mean of *y*, ${\hat{y}}_{ijk}$ represents the yield of genotype *i*, environment *j* and replicate *k*, $G_{i}$ and $E_{j}$ the estimated fixed effects of genotype and environment in S. Error term $GE_{ijk}∼N(0,\sigma^{2})$ then represents the residual genotype-by-environment interaction of the sample. We note that (3) represent a minimal decomposition. Nonetheless, we deliberately estimate $G_{i}$ and $E_{j}$ as fixed effects to avoid the adverse effects of shrinkage in downstream modeling (Holland and Piepho 2024).

Next, we utilize the fitted effects ${\hat{G}}_{i}$, ${\hat{E}}_{j}$ and residual ${\hat{GE}}_{ijk}$ from (3) as 3 sets of training labels to approximate mappings $f_{g}$, $f_{e}$ and $f_{ge}$ from genetic features $x_{i}^{g}$ and environmental features $x_{j}^{e}$:


(4a ) $${\hat{G}}_{i}={\hat{\;f}}_{g}(x_{i}^{g}),$$

(4b ) $${\hat{E}}_{j}={\hat{\;f}}_{e}(x_{j}^{e}),$$

(4c ) $${\hat{GE}}_{ijk}={\hat{\;f}}_{ge}(x_{i}^{g},x_{j}^{e}),$$

with ${\hat{\;f}}_{g}$, ${\hat{\;f}}_{e}$ and ${\hat{\;f}}_{ge}$ being 3 unique DNNs that are optimized separately. This effectively decomposes the prediction task into 3 component tasks: (4a) is a standard genomic prediction task, (4b) a standard enviromic prediction task, and (4c) a new interaction prediction task. The implementation details for each prediction task are described in *Model implementation* below. In order to integrate the learned representations from genomic prediction and enviromic prediction into interaction prediction, we initialize the parameters $\theta_{ge}$ of ${\hat{\;f}}_{ge}$ with the learned weights $\theta_{g}$ and $\theta_{e}$ of ${\hat{\;f}}_{g}$ and ${\hat{\;f}}_{e}$:


(5) $$(\;\theta_{g},\;\theta_{e},\;\theta_{x}^{\left(0\right)}\;)\;\underset{⟶}{\mathrm{init}}\;\;\theta_{ge}^{\left(0\right)},$$

where the parameters of the interaction component of ${\hat{\;f}}_{ge}$, $\theta_{x}$ are initialized using any standard initialization. To predict *y* from genetic and environmental features for new genotypes and environments, one can finally substitute (4a), (4b) and (4c) in (3) to obtain


(6) $${\hat{y}}_{ij}=\hat{\mu }+{\hat{\;f}}_{g}(x_{i}^{g})+{\hat{\;f}}_{e}(x_{\;j}^{e})+{\hat{\;f}}_{ge}(x_{i}^{g},x_{\;j}^{e}),$$

which directly estimates yield *y* for genotype *i* and environment *j* from genetic features $x_{i}^{g}$ and environmental features $x_{j}^{e}$. Together, (2)–(6) form our proposed method, the Structured Interaction Neural Network (SINN). SINN utilizes the known structure of the training set S to decompose $y_{s}$ into independent label sets for genetic ($G_{i}$), environmental ($E_{j}$) and interaction effects ($GE_{ijk}$). This enables SINN to avoid the adverse effects of multimodal optimization of DNNs, while still utilizing all information in the prediction of genotype-environment interactions.

### Datasets

Two datasets were utilized in this study: the Genomes to Fields 2022 Maize GxE prediction competition data (G2F2022) (Lima et al. 2023) was used as primary dataset for in-depth model intercomparison and interpretation, while the MaizeGEP dataset (D Zhang et al. 2026) was used to assess the general applicability of SINN using independent data.

#### Genomes to fields

The G2F2022 dataset comprises data from a North American maize multi-environment trial conducted by the Genomes to Fields project (Lawrence-Dill et al. 2019). This dataset was curated and shared for the 2022 Maize GxE prediction competition (Washburn et al. 2025). The competition challenged participants to predict the plot yields of 505 new and 43 previously observed maize hybrids in 26 environments across North America for the year 2022, based on a training set of the plot yields of 4,683 maize hybrids in 217 environments across 47 locations in the years 2014–2021. The complete dataset included over 140,000 measured plots, from which 10,290 plots are assigned to the test set.

The dataset included genetic markers, weather and soil measurements, management information and plot yields. For each hybrid, information on 437,214 single- nucleotide polymorphisms (SNPs) was provided. For each environment 29 soil features, 5 management features and 16 daily weather features were provided. Each plot record included planting date, harvest date and planting density. For a complete description of the dataset, we refer to Lima et al. (2023).

The training dataset was filtered and imputed to ensure each training sample included a full set of features. Training samples without plot yields, genotypes without SNPs and trials without weather data were removed, leaving 123,517 samples for 4,417 genotypes and 212 environments, across 47 locations in 8 years. All features with a higher than 30% rate of missing values were removed. SNPs with a minor allele frequency below 1% or with more than 10% missing values were removed, and the resulting SNPs were randomly downsampled to 20,000 SNPs for the DNN-based methods. Imputation methods varied per feature, as follows: SNPs were imputed with the most frequent genotype at each locus. Management features consisted of 2 categorical features, representing whether the environment was irrigated and was a disease trial. For each, missing values were imputed with the most common class. Soil features were imputed by nearest spatial neighbor across training environments. Weather features within a growing season were imputed by linear interpolation in time. For each plot, weather features were aligned from 7 d before sowing to 133 days after sowing, giving a time series of 140 d for each plot. Missing values for planting date, harvest date and planting density were imputed with means within each environment where available, and global means elsewhere. The final preprocessed dataset retained 20,000 genetic markers, 11 daily weather features over 140 days, 20 soil features and 2 management features.

#### MaizeGEP

As secondary dataset we utilized the MaizeGEP dataset (D Zhang et al. 2026), which contains records of 260 hybrid maize varieties and 2,382 trials, spanning 2017–2023, sourced from the Chinese National Maize Variety Regional Trials. For each hybrid, information on 12,233 SNPs was provided. For each environment 11 daily weather features, no soil features and no management features were provided. In place of soil features, we use latitude and longitude values. Corrected BLUEs per genotype-environment combination were provided for 11 phenotypic traits, based on a randomized complete block design with 3 replicates per combination. Yield was used as the target trait in this study, for which 42,975 BLUEs were included. For specification on data preprocessing and in-depth analysis of the dataset we refer to the original work (D Zhang et al. 2026).

### Experiments

We investigate the accuracy, biological interpretability and general applicability of the proposed SINN framework in 4 experiments. *Experiment 1 and 2* measure the accuracy of SINN and its component models using the G2F2022 dataset. An overview of our experimental framework for *Experiment 1 and 2* is shown in Fig. 2. We split the dataset into 2 subsets: a cross-validation set (years 2014–2021) and a holdout test set (final year 2022). The cross-validation set was used for all statistical decompositions, model training and model cross-validation. The holdout test set was used only to evaluate trained models, and was identical to the test set used in the competition. *Experiment 3* utilizes SINN models trained in *Experiment 2* and provides an interpretation of the importance of genetic and environmental features, and interactions between them. Finally, *Experiment 4* replicates *Experiment 2* on the MaizeGEP dataset to provide an assessment of the general applicability of SINN.

**Fig. 2.**
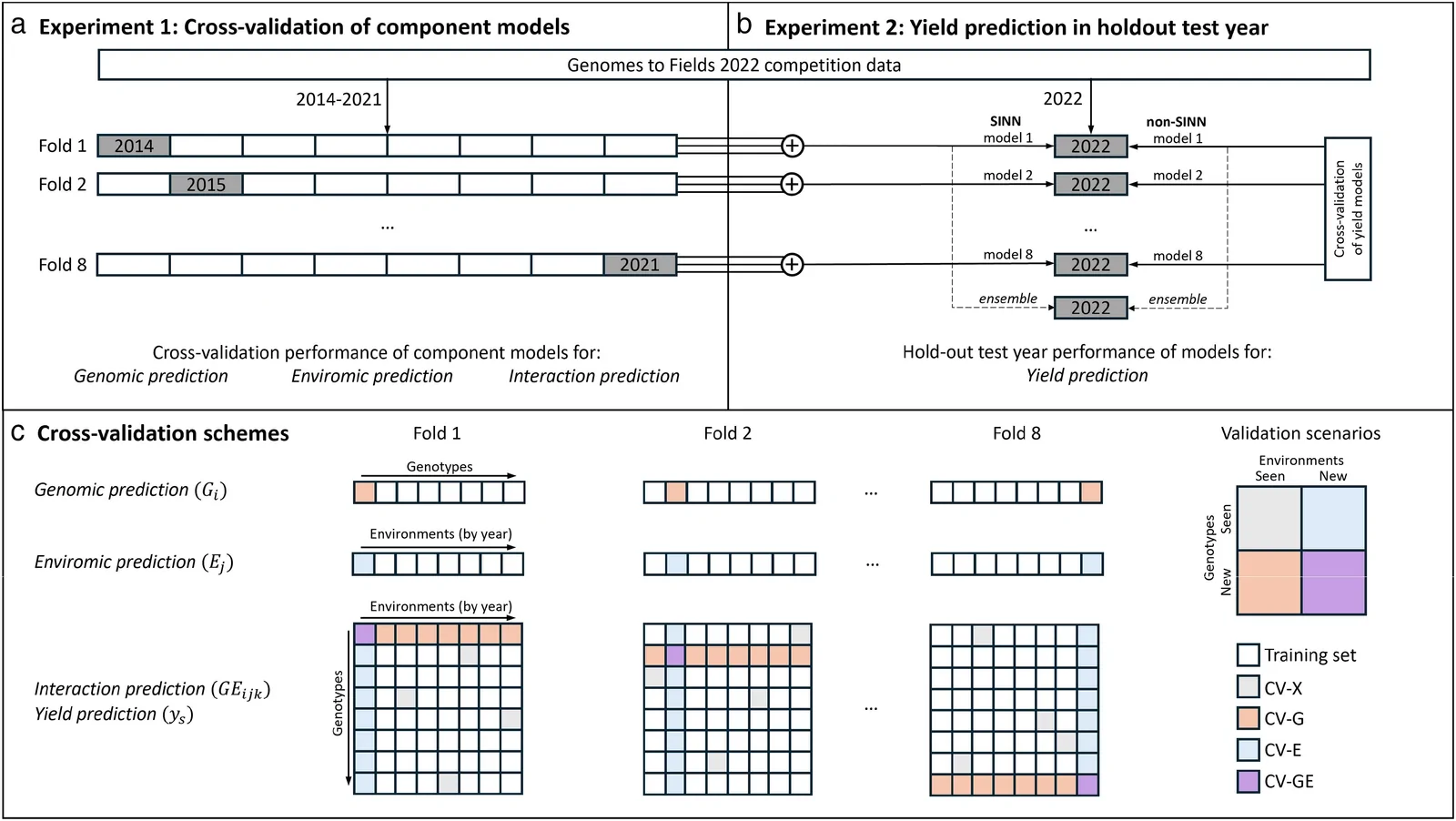
Overview of experiments and cross-validation schemes implemented on the Genomes to Fields data in this work. a) *Experiment 1*. In the cross-validation set (years 2014–2021), we performed eight-fold cross-validation of component models for genomic prediction, enviromic prediction and interaction prediction. b) *Experiment 2*. We evaluated yield prediction models in the holdout test set (year 2022), measured stability in test set performance over cross-validation training sets (model 1-8), and measured performance of averaged predictions (ensemble). SINN models were constructed through summation of component models from *Experiment 1*, non-SINN models were fitted directly on yield. c) Cross validation schemes and scenarios for all modeling tasks defined in *Experiment 1 and 2*.

### Experiment 1: cross-validation of component models

In our method, we proposed to model unimodal (genetic, environmental) and multimodal (interaction) components of yield separately. This created the opportunity to evaluate modeling approaches per component task. We applied the decomposition in (3) to the cross-validation set (years 2014–2021) and obtained unimodal genetic targets $G_{i}$, unimodal environmental targets $E_{j}$ and residual genotype-environment interaction targets $GE_{ijk}$. For each type of component task with corresponding target, we evaluated a set of models across 8 cross-validation folds. The set of component models used per component target are further described in section *Models*.

As illustrated in Fig. 2c, different cross-validation schemes were used for each component task. Genomic prediction of $G_{i}$ was evaluated on new genotypes, corresponding to scenario CV-G. Each validation fold for CV-G contained $\frac{1}{8}$ of genotypes. Enviromic prediction of $E_{j}$ was evaluated on new environments, corresponding to scenario CV-E. We split environments based on a single year per validation set, again obtaining 8 folds.

For interaction prediction of $GE_{ijk}$, we defined a cross-validation scheme with 4 scenarios. For each fold, $\frac{1}{8}$ of genotypes and 1 yr of environments were selected, corresponding to the same validation genotypes and environments used for $G_{i}$ and $E_{j}$. These settings formed cross-validation scenarios CV-G and CV-E. Their intersection formed scenario CV-GE, predicting interactions for new genotypes and environments. Finally, we obtained a fourth validation scenario by sampling random combinations of observed genotypes and observed environments, and holding out all replicates of these combinations in the validation fold. This reflects scenario CV-X, new interactions of observed genotypes and environments. We sampled 2,000 interactions for each fold of CV-X. Model performance in scenario CV-GE was used as target criterion for model and hyperparameter selection, as it reflects most closely the scenario of the holdout test set.

### Experiment 2: yield prediction in holdout test year

In order to evaluate the effectiveness of the proposed SINN method, we conducted a second experiment that mirrored exactly the test conditions of the Genomes to Fields 2022 competition (Washburn et al. 2025). We evaluated a set of models on a holdout test set (year 2022), comprising of 43 observed and 505 new genotypes grown in 26 new environments. All environments were in repeated locations from previous years, but had new weather conditions.

Models were evaluated in their capacity to predict maize yields (Mg/ha) of genotype-environment combinations. The competition reported a single target yield value per combination, which we infer is an average over an unknown number of replicates. We constructed the complete SINN model from component SINN models evaluated in *Experiment 1*, following (6). This is shown in Fig. 2b as summation of 3 component models per cross-validation fold.

Non-SINN models (further specified in *Models*) were not constructed from component models in *Experiment 1*. Instead, they were directly fitted on observed plot yields in the cross-validation set. In order to correctly compare variability in model errors between SINN and non-SINN methods, we fitted 1 model of each non-SINN model type per cross-validation fold, with the same cross-validation scheme used for interaction prediction. Again, cross-validation scenario CV-GE was used for model selection and hyperparameter optimization.

Using the models obtained through cross-validation would have underestimated the potential performance of models on the holdout test set, as each model was trained with a subset of all available samples. However, it is highly informative of stability of modeling approaches across dataset subsets. To retain this information on stability while avoiding under-estimation of model performance, we reported both predictions of individual models and ensembled predictions per model. We reported performance of individual models when comparing models which we implemented in this study, but reported ensembled predictions when comparing with current state-of-the-art from Washburn et al. (2025). For each model type, we ensembled predictions for the hold-out test set over the set of 8 models obtained through cross-validation.

### Experiment 3: interpretation of predicted biological relationships

To further validate the quality of yield predictions by SINN, we investigate the feature importance and feature interactions learned by the model across *Experiment 1 and 2*. Both are calculated over all test samples of the G2F2022 dataset, using a single ensemble of 8 SINN models (1 per cross-validation fold). For overall feature importance we calculate SHapley Additive exPlanations (SHAP) values of individual features, which provide an additive measure of the average contribution of each feature to yield across the test set (Lundberg and Lee 2017). We quantify pairwise interaction strength between genetic markers and environment features with Integrated Hessians (IH) (Janizek et al. 2021), which measure feature-feature interactions by integrating the model’s Hessian along a path from baseline input to each test sample.

### Experiment 4: yield prediction in national variety trials

We replicated *Experiment 2* on the MaizeGEP dataset to investigate the external generalization of the SINN framework. The final year of data (2023) was used as holdout test set, comprising of 3,538 yield values of 31 observed and 7 new genotypes grown across 325 new environments. The previous years (2017–2022) were used in six-fold cross-validation of model components. We follow a simplified implementation of the cross-validation schemes of *Experiment 1 and 2*, which we document in Appendix A.

### Metrics

As main evaluation metrics we used the root mean squared error (RMSE), mean absolute error (MAE) and the Pearson correlation coefficient (*r*). The mean squared error (MSE) was used as optimization criterion. To compare prediction of genetic versus environmental signals, we define the *genetic accuracy* as average within-environment correlation, calculated as following:


(7) $${\bar{r}}_{g}=\frac{1}{J}\sum_{\;j=1}^{J}r_{j}\left(y,\hat{y}\right),$$

Similarly, we define the *environmental accuracy* as average within-genotype correlation:


(8) $${\bar{r}}_{e}=\frac{1}{I}\sum_{i=1}^{I}r_{i}\left(y,\hat{y}\right),$$

### Models

We used a similar approach for fitting each component of SINN, ${\hat{\;f}}_{g}$, ${\hat{\;f}}_{e}$ and $\hat{\;f_{ge}}$. The models were fitted with the AdamW optimizer (Loshchilov and Hutter 2019), minimizing MSE as training objective. We linearly increased the learning rate from zero to the chosen maximum learning rate over the first 5 training epochs of each model. The number of training epochs for each task was determined by manual experimentation prior to hyperparameter tuning. For the G2F2022 dataset, the final validation fold was used for hyperparameter tuning of each model. For environments, this fold corresponded to the trials conducted in 2021. The search space and number of iterations per model of the hyperparameter selection are described in Table A1 in Appendix A. We report final hyperparameters selected for the G2F2022 dataset below. For the MaizeGEP dataset we further improved the stability of hyperparameter selection, including selection of optimal number of training epochs across all cross-validation folds. For details on hyperparameter tuning and final hyperparameters selected for the MaizeGEP dataset, we refer to Table A2 in Appendix A.

As genomic prediction model ${\hat{\;f}}_{g}$ we used a multi-layer perceptron (MLP) (Rosenblatt 1958). Input SNPs were encoded as the count of alternate alleles minus 1 (-1, 0 or 1), resulting in a genetic input dimension $d_{g}$ of 20,000. The MLP used had 3 layers: 1 input layer, 1 hidden layer and 1 output layer with 96, 48 and 1 nodes respectively. Each input and hidden layer was followed by a layer normalization (Ba et al. 2016) and dropout layer (dropout rate 0.5) (Srivastava et al. 2014), then an activation function. We used a ReLu layer (Nair and Hinton 2010) as activation function. Before the output layer, a sigmoid activation function was used instead. We trained ${\hat{\;f}}_{g}$ for 250 epochs, with a batch size of 256, a learning rate of 0.01 and a weight decay of $1\times 10^{-4}$.

As enviromic prediction model ${\hat{\;f}}_{e}$ we used the same base architecture as ${\hat{\;f}}_{g}$. Daily weather features were averaged over the growing season and concatenated with soil and management features, resulting in an environmental input dimension $d_{e}$ of 33. The model had 1 input layer, 2 hidden layers and 1 output layers with 64, 64, 32 and 1 nodes respectively. We trained ${\hat{\;f}}_{e}$ for 500 epochs, with a batch size of 32, a learning rate of 0.001 and a weight decay of $3\times 10^{-5}$. We noted that the initial performance of ${\hat{\;f}}_{e}$ during hyperparameter tuning was unstable and resulted in models with a low Pearson Correlation Coefficient, but low error. This indicated selection for models which capture the validation set mean yield, but not structure. To rectify this, we added a penalty of −5r to the criterion, resulting in a pragmatic selection criterion of MSE−5r with approximately equally scaled MSE and *r*.

As interaction prediction model, we reused the trained ${\hat{\;f}}_{g}$ and ${\hat{\;f}}_{e}$ as encoder networks. The output of the final linear hidden layer of each encoder was projected with a learned linear projection to an embedding vector of size 64. To limit our model to first-order interactions, the genetic and environment embeddings were fused using a dot product operation to obtain predicted yield values. Training consisted of learning the linear projection weights and finetuning the parameters of the encoder networks. We trained the model for 250 epochs, with a batch size of 256, a learning rate of 0.01 and a weight decay of $3\times 10^{-3}$. In this work, we denote all models that are constructed using ${\hat{\;f}}_{g}$, ${\hat{\;f}}_{e}$ and ${\hat{\;f}}_{ge}$ with *SINN*, as they were constructed through the SINN framework. However, we note that models that exclude ${\hat{\;f}}_{ge}$ do not allow for interactions, making the *I* superfluous. Nonetheless, we define the following set of SINN models:


$$\begin{array}{rl} \text{G-SINN}:\;\;\;y_{ijk} & =\hat{\mu }+{\hat{\;f}}_{g}(x_{i}^{g})+\epsilon_{ijk},\\ \text{E-SINN}:\;\;\;y_{ijk} & =\hat{\mu }+{\hat{\;f}}_{e}(x_{\;j}^{e})+\epsilon_{ijk},\\ \text{G+E-SINN}:\;\;\;y_{ijk} & =\hat{\mu }+{\hat{\;f}}_{g}(x_{i}^{g})+{\hat{\;f}}_{e}(x_{\;j}^{e})+\epsilon_{ijk},\\ \text{G}\times \text{E-SINN}:\;\;\;y_{ijk} & =\hat{\mu }+{\hat{\;f}}_{g}(x_{i}^{g})+{\hat{\;f}}_{e}(x_{\;j}^{e})+{\hat{\;f}}_{ge}(x_{i}^{g},x_{\;j}^{e})+\epsilon_{ijk}, \end{array}$$

Throughout the results, G×E-SINN and SINN are used interchangeably. Additionally, SINN-based methods without ${\hat{\;f}}_{ge}$ (G-SINN, E-SINN, and G+E-SINN) should be understood as ablations of the SINN framework, not as separate modeling approaches.

### Baseline models

As baseline models, we used the winning model and the highest ranking DNN of the G2F2022 dataset competition (Washburn et al. 2025). Additionally, we fitted a set of commonly utilized statistical reaction-norm-based models that extend G-BLUP (Meuwissen et al. 2001) with an environmental covariance matrix and interaction matrix based on genetic markers and environmental covariates (Jarquín et al. 2014). In contrast to the models fitted in the original work, we did not include separate (unstructured) effects for lines and trials, to avoid absorption of effects from marker and environmental covariate-based terms. We define this set of models as following:


$$\begin{array}{rl} \text{G-BLUP}:\;\;y_{ijk} & =\mu +g_{i}+\epsilon_{ijk},\\ \text{E-BLUP}:\;\;y_{ijk} & =\mu +e_{j}+\epsilon_{ijk},\\ \text{G+E-BLUP}:\;\;y_{ijk} & =\mu +g_{i}+e_{j}+\epsilon_{ijk},\\ \text{G}\times \text{E-BLUP}:\;\;y_{ijk} & =\mu +g_{i}+e_{j}+ge_{ij}+\epsilon_{ijk}, \end{array}$$

with components defined as following:


$$\begin{array}{rl} g_{i} & ∼N(0,\sigma_{g}^{2}\Sigma_{g}),\;e_{j}∼N(0,\sigma_{e}^{2}\Sigma_{e}),\\ ge_{ij} & ∼N(0,\sigma_{ge}^{2}\Sigma_{g}⊗\Sigma_{e}),\;\epsilon_{ijk}∼N(0,\sigma_{\epsilon }^{2}I),\\ \Sigma_{g} & =\frac{x^{g}{x^{g}}^{⊤}}{tr(x^{g}{x^{g}}^{⊤})/n_{g}},\;\Sigma_{e}=\frac{x^{e}{x^{e}}^{⊤}}{tr(x^{e}{x^{e}}^{⊤})/n_{e}}, \end{array}$$

where again $y_{ijk}$ denotes yield for genotype *i*, environment *j* and replicate *k*, $g_{i}$ is the predicted genomic effect, $e_{j}$ the predicted effect of the environment, and $ge_{ij}$ the predicted effect of the genotype-environment interaction. $\Sigma_{g}$ was the genomic relationship matrix of $n_{g}$ genotypes, $\Sigma_{e}$ the environmental relationship matrix of $n_{e}$ environments, calculated from genetic features $x^{g}$ and environmental features $x^{e}$ respectively. For *Experiment 2*, we fitted each BLUP-based model on yield directly. Memory requirements for $\Sigma_{g}⊗\Sigma_{e}$ scaled quadratically with number of samples. As our experiments were constrained to 500GB of RAM, we were forced to drop replicates and entries for the highest frequency genotypes until 40% of the samples remained when fitting G×E-BLUP. To keep implementation consistent between BLUP-based models, we used the same downsampling fraction as G×E-BLUP for other BLUP-based models fitted on yield. Initial experiments indicated that predictive performance of all BLUP-based methods saturated at downsampling fractions below 40%. As such, we would expect no increase in performance if the downsampling fraction were increased beyond 40%. For *Experiment 1*, we fitted 1 BLUP-based model on each set of labels: we fitted G-BLUP on $G_{i}$, E-BLUP on $E_{j}$, and G×E-BLUP on $GE_{ijk}$. Again, we dropped replicates from $GE_{ijk}$ until 40% of samples remained. For *Experiment 4*, all training samples were used to fit the BLUP-based models.

As additional DNN baseline, we replicated a recent DNN-based model by Kick et al. (2023) that was developed using a subset of the G2F2022 dataset. We only tuned the learning rate and weight decay. The model architecture and other hyperparameters remained unchanged. The model consisted of a genetic encoder, a soil encoder, a weather encoder and an interaction module. The genetic encoder was an MLP with 1 hidden layer using principal components of $x^{g}$ as inputs. The weather encoder was a Convolution Neural Network (CNN) taking daily weather values as inputs. The soil encoder was an MLP with 1 hidden layer taking the soil and management values as input. The interaction module took the concatenated outputs of the final hidden layer of each encoder as input and was an MLP with 5 hidden layers. For further details, we refer to the original implementation (Kick et al. 2023).

For the first set of experiments, we trained only the relevant DNN components, adding a linear output layer if not present. We trained the genetic encoder on $G_{i}$ for 250 epochs, with a batch size of 256, a learning rate of $3\times 10^{-4}$ and a weight decay of $1\times 10^{-4}$. We jointly trained both the weather and soil encoders on $E_{j}$ for 500 epochs, with a batch size of 32, a learning rate of $1\times 10^{-3}$ and a weight decay of $3\times 10^{-4}$. To predict $GE_{ijk}$, we followed the same procedure as the SINN model, and initialized the full DNN with the trained encoder weights. Then, we trained the model on $GE_{ijk}$ for 250 epochs, with a batch size of 192, a learning rate of $3\times 10^{-3}$ and a weight decay of $3\times 10^{-3}$. For the second set of experiments, we trained the full model with yield as target variable on CV-GE. Following hyperparameter tuning, we trained for 150 epochs with a batch size of 192, a learning rate of 0.01 and a weight decay of $3\times 10^{-3}$.

## Results

In this section, we provide a stagewise analysis of the proposed SINN framework. Following the modeling steps of SINN (decomposition, component modeling, structured prediction), we focus on answering the following 4 questions:

**Preliminary:** How is the variance in yield partitioned among genotypes, environments and genotype-environment interactions?
**Experiment 1:** How do different types of models predict isolated unimodal and multimodal components of yield?
**Experiment 2:** How well does SINN predict maize yield in the next year of a multi-environment trial, in comparison to current state-of-the-art?
**Experiment 3:** What biological relationships between SNPs, ECs and yield are modelled by SINN?
**Experiment 4:** Can SINN generalize to yield prediction in a second, independent dataset?

### Preliminary: analysis of variance

As SINN relies on a statistical decomposition of yield across genotypes, environments and genotype-environment interactions, we preclude our results with an analysis of variance for the data used in model fitting and cross-validation (years 2014–2021). The current implementation of SINN utilizes an additive decomposition. We use a different, more informative decomposition to investigate the variance of yield across the training data. We fit a three-way random effect ANOVA between genotype, year and location, which is shown in Table 1.

**Table 1.** Three-way ANOVA of yield by genotype (G), location (L) and year (Y) for cross-validation set spanning 2014–2021.

Component		% Variance	% Total
Genotype	G	5.2	5.2
Environment	L	25.0	59.5
	Y	9.1	
	L x Y	25.4	
Interaction	G x L	1.8	9.2
	G x Y	1.4	
	G x L x Y	6.0	
Residual	ϵ	26.2	26.2

Genotype, environment and interaction components contributed 5.2%, 59.5% and 9.2% to overall variance. The location effect contributed 42% of the environmental variance, suggesting a considerable location-specific component to environmental effects. Finally, the residual variance was 2.85× the genotype-by-environment variance, indicating that the genotype-by-environment specific signal is challenging to separate from the residual within-environment noise.

### Experiment 1: cross-validation of model components

We now consider the performance of component models in predicting the decomposed yield components ${\hat{G}}_{i}$, ${\hat{E}}_{j}$ and ${\hat{GE}}_{ijk}$ for cross-validation folds across the S. An overview of the results of each component is shown in Fig. 3, with colors matching model types (BLUP-based, vanilla DNN, and SINN). For reference, cross-validation schemes per component task were provided in Fig. 2c.

**Fig. 3.**
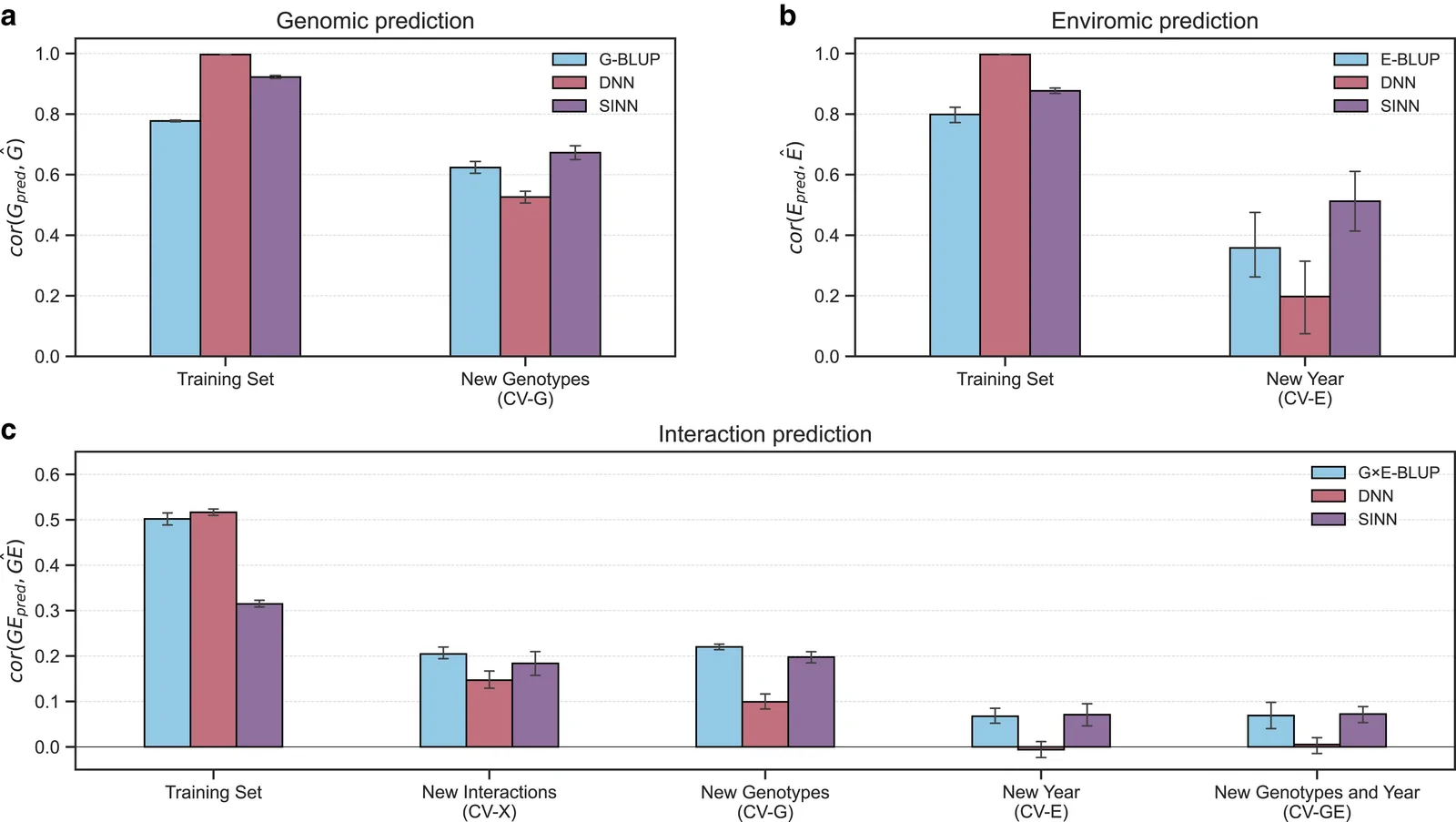
Predictive performance in the G2F2022 dataset of SINN component models (purple) compared to BLUP-based (blue) and DNN (red) component models. Error bars show standard deviation over 8 cross-validation folds. a) Predictive ability for genomic prediction of the estimated effect of new genotypes (CV-G). b) Predictive ability for enviromic prediction of new environments (CV-E). c) Predictive ability for interaction prediction of residual genotype-by-environment interaction in scenarios from left to right: training set, new interactions of observed genotypes and environments (CV-X), new genotypes and observed environments (CV-G), observed genotypes and new environments (CV-E), and new genotypes and environments (CV-GE).

First, the genomic prediction results for genetic effects ${\hat{G}}_{i}$ of new genotypes (CV-G) are shown in Fig. 3a. The genetic encoder of SINN (MLP) had the highest cross-validation predictive ability, followed by G-BLUP and the genetic encoder of DNN (principal component-based MLP). Furthermore, the baseline DNN had a correlation of near 1 on the training set, indicating severe overfitting.

We show the enviromic prediction results of environment effects ${\hat{E}}_{j}$ in new environments (CV-E) in Fig. 3b. Overall, the cross-validation predictive ability was lower than for genomic prediction and showed a larger variance over folds. The enviromic encoder of SINN (MLP) had the highest performance, followed by E-BLUP and the environment encoder of the DNN (CNN). The baseline DNN had a near-perfect training correlation, again indicating severe overfitting.

Finally, the prediction of residual genotype-by-environment interactions ${\hat{GE}}_{ijk}$ is shown across 4 validation scenarios in Fig. 3c. The performance of all models was highest when predicting new interactions for observed hybrids and observed trials (CV-X). Surprisingly, G×E-BLUP and SINN showed no decrease in predictive ability when predicting for new genotypes (CV-G) compared to CV-X, indicating strong generalization of performance to new genotypes. The DNN dropped in performance from an predictive ability of 0.15 to 0.10 for new genotypes, indicating overfitting of interactions on observed genotypes.

All 3 models showed a large drop in performance when predicting for new environments (CV-E) compared to CV-X, indicating poor generalization of interaction prediction to new environments. On the cross-validation scenario reflecting the G2F2022 test scenario, predicting interactions for new genotypes and environments (CV-GE), SINN and G×E-BLUP had a similar predictive ability of 0.07, while the baseline DNN had a predictive ability of 0.01.

For all models, the predictive ability of CV-GE was similar to CV-E, but considerably lower than CV-G, indicating that the low reported accuracies for CV-GE are primarily driven by poor generalization to new environments. Furthermore, the three-way ANOVA decomposition in Table 1 can be used to estimate the predictive ability of a perfect interaction prediction model. The interaction-specific variance divided by the total interaction plus residual variance is estimated as 9.2/35.4≈0.26. As such, we can roughly attribute 26% of the variance in ${\hat{GE}}_{ijk}$ to genotype-environment interactions. Converting from explained variance to correlation, we obtain a maximum achievable correlation of $\sqrt{9.2/35.4}\approx 0.51$ for a model that perfectly predicts the genotype-environment interactions for each genotype-environment combination. Both G×E-BLUP and DNN reached this upper bound on the training set, which again indicates overfitting. Furthermore, this irreducible error lowered the observed predictive ability of all interaction prediction models across all scenarios, as it includes the accuracy of predicting residual noise.

Overall, SINN exhibited lower overfitting and better generalization across all tasks and scenarios when compared to the baseline DNN components. SINN showed the largest improvements over BLUP-based models in predicting ${\hat{E}}_{j}$, a smaller improvement in predicting ${\hat{G}}_{i}$ and a comparable performance in predicting ${\hat{GE}}_{ijk}$. Interaction prediction was the most challenging task out of the 3 component tasks, followed by enviromic prediction and genomic prediction. Difficulty of interaction prediction scenario followed the same trend, with lower predictive abilities reported for prediction in new environments (CV-E) than for new genotypes (CV-G) across all models.

### Experiment 2: maize yield prediction in holdout test year of a multi-environment trial

In the previous section, we observed that SINN outperforms other models on component modeling tasks. Next, we investigate whether this leads to an increased predictive ability for the prediction of maize yield in a holdout test year of a multi-environment trial. To this end, we compare SINN with other models fitted directly on yield, and with previously reported models in the The Genomes to Fields 2022 Maize Genotype by Environment Prediction Competition (Washburn et al. 2025).

In Fig. 4a the performance of all 3 classes of models is shown in predicting maize yield in the holdout test year (2022) of the G2F maize hybrid dataset. We compare 4 statistical models including G-BLUP (Meuwissen et al. 2001) and 3 extensions including environmental covariates (E-BLUP, G+E-BLUP) and genotype-by-environment interactions (G×E-BLUP) following Jarquín et al. (2014). As baseline Deep Neural Network (DNN), we use the network architecture from Kick et al. (2023). We report on 4 variants of the SINN model: a genetic model component (G-SINN), an environment model component (E-SINN), an additive combination of the genetic and environmental model components (G+E-SINN), and a full model with genetic, environment and genotype-by-environment interaction (SINN). SINN had a higher and more consistent predictive ability (0.59±0.03) than DNN (0.38±0.07) and G×E-BLUP (0.39±0.07), which had approximately equal predictive ability. G-BLUP (0.15), E-BLUP (0.28) and G+E-BLUP (0.34) had a lower predictive ability than G-SINN (0.24), E-SINN (0.53) and G+E-SINN (0.58) respectively.

**Fig. 4.**
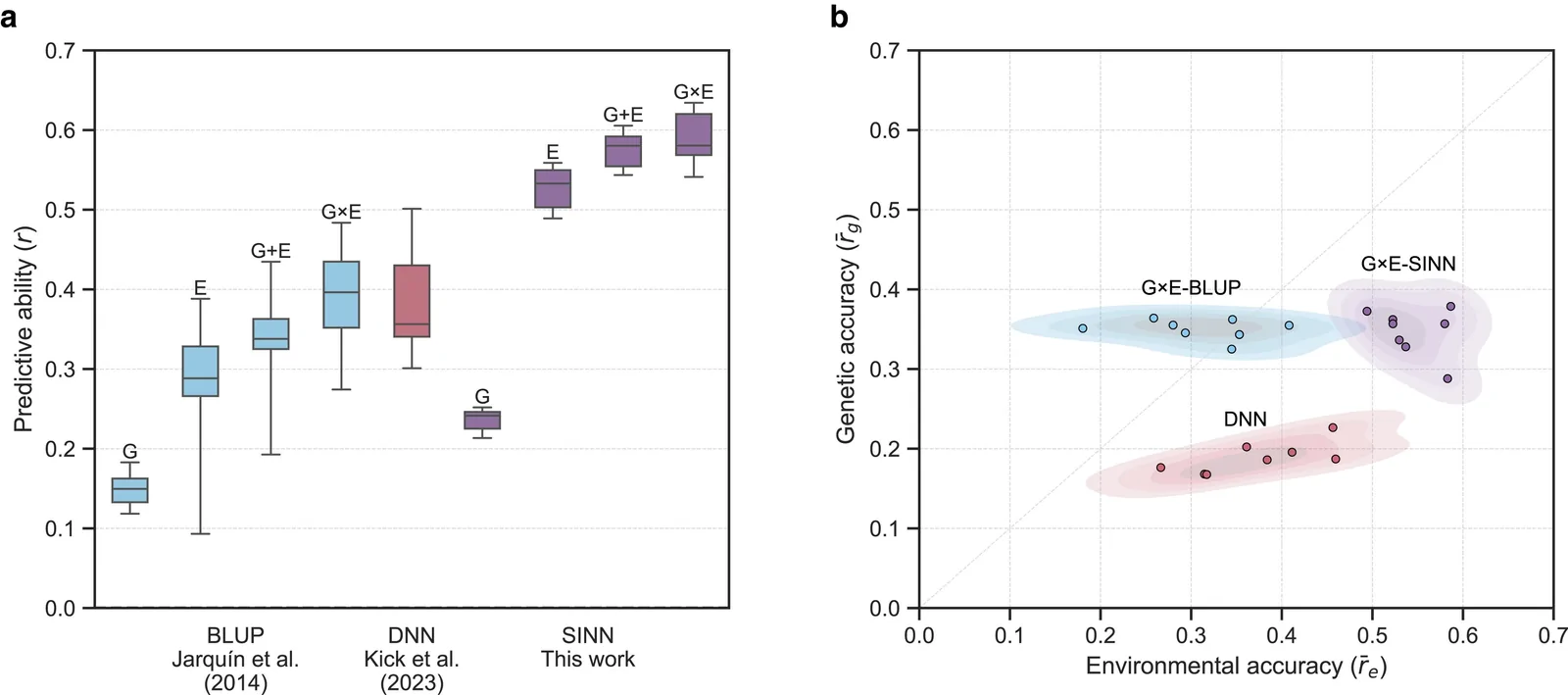
Predictive performance for plot-level maize yield in test year (2022) of Genome to Fields, including unobserved hybrids. a) Overall predictive ability (Pearson Correlation Coefficient) of models fitted in 8 cross-validation folds. b) Kernel density estimate of correlation per genotype and per environment of the same 8 models for G×E-BLUP (blue), DNN (red) and SINN (purple).

Dissecting overall predictive ability into genetic and environmental accuracies reveals qualitative differences in the predictions of BLUP-based, DNN-based and SINN-based models. Genetic and environmental accuracies, defined in (7) and (8), for the 3 models types with genotype-by-environment interactions are shown in Fig. 4b. While G×E-BLUP and DNN had similar overall predictive ability, DNN had a significantly lower genetic accuracy (0.19) than G×E-BLUP (0.35), indicating it performed worse at predicting the relative performance of genotypes within each environment. DNN had a higher environmental accuracy (0.37) compared to G×E-BLUP (0.31). SINN matched G×E-BLUP in genetic accuracy (0.35), while improving over both G×E-BLUP and DNN in terms of environmental accuracy (0.54). Overall, SINN improves over DNN in terms of genetic and environmental accuracies, and improves over G×E-BLUP in terms of environmental accuracy. Table 2 directly compares the 3 models with the competition results from Washburn et al. (2025). To keep models and data splits consistent across experiments, we did not fit a model using the full training and validation. Instead, we ensembled the predictions of models fitted on each cross-validation fold by direct averaging to obtain model predictions that incorporate information of the full training and validation set. Table 2, further reports average performance and 95% confidence interval across 10 replicates of each model per scenarios within the test set: New Year (CV-E), and New Genotypes and Year (CV-GE).

**Table 2.** Predictive performance across test scenarios. Competition scenario comprises the full test set, composed of 6% of samples with new environments in a new year, and 94% with both new genotypes and new environments in a new year. Performance of competition models (Washburn et al. 2025) are represented by a single score. Performance metrics of G×E-BLUP (Jarquín et al. 2014), DNN (Kick et al. 2023) and SINN (this work) are calculated by ensembling predictions of 8 models, each trained with 1 cross-validation fold. Scores are shown as *Average [95% Confidence Interval]*, calculated over 10 replicated ensembles. Best model per column and scenario is shown in **bold**, with * denoting significant difference to second-best model (Wilcoxon signed-rank test, p<0.05). Literature scores from Washburn et al. (2025) are treated as fixed references due to lack of reported replicates or confidence intervals. Reported metrics are RMSE and MAE in Mg/ha, and Pearson correlation coefficient calculated across all samples (*r*) or within environments (${\bar{r}}_{g}$).

Scenario	Model	RMSE ↓	MAE ↓	*r* ↑	${\bar{r}}_{g}$ ↑
Competition	Competition best (CLAC)	2.46	1.93	**0.63**	0.36
	Competition best DNN (SmAL)	2.52	2.00	0.59	0.15
	G×E-BLUP	2.90 [2.89, 2.91]	2.35 [2.34, 2.36]	0.43 [0.42, 0.43]	0.38 [0.38, 0.38]
	DNN	2.85 [2.79, 2.90]	2.29 [2.23, 2.35]	0.47 [0.45, 0.49]	0.25 [0.24, 0.27]
	SINN	2.40 [2.37, 2.43]*	1.85 [1.83, 1.88]*	0.63 [0.61, 0.64]	0.38 [0.38, 0.39]*
New Year (6%)	G×E-BLUP	2.79 [2.78, 2.80]	2.26 [2.25, 2.26]	0.46 [0.45, 0.46]	0.60 [0.60, 0.60]*
	DNN	2.76 [2.71, 2.80]	2.16 [2.14, 2.18]	0.54 [0.52, 0.55]	0.55 [0.54, 0.56]
	SINN	2.62 [2.59, 2.64]*	2.00 [1.98, 2.02]*	0.59 [0.58, 0.61]*	0.59 [0.58, 0.59]
New Genotypes and Year (94%)	G×E-BLUP	2.91 [2.90, 2.91]	2.36 [2.35, 2.37]	0.41 [0.41, 0.42]	0.31 [0.31, 0.32]
	DNN	2.85 [2.79, 2.91]	2.30 [2.23, 2.37]	0.46 [0.44, 0.48]	0.17 [0.16, 0.18]
	SINN	2.38 [2.36, 2.41]*	1.84 [1.82, 1.87]*	0.62 [0.61, 0.63]*	0.34 [0.34, 0.34]*

SINN obtained the lowest RMSE, MAE and highest genetic accuracy (${\bar{r}}_{g}$) of all models in the competition setting. Similar to DNN, the competition best DNN had very low genetic accuracy, indicating that state-of-the-art DNNs for genomic prediction in new environments suffer do not predict genetic differences as well as BLUP- or SINN-based methods. SINN outperformed G×E-BLUP and DNN across both New Year (CV-E) and New Genotypes and Year (CV-GE) scenarios for RMSE, MAE and *r*. G×E-BLUP obtained a higher genetic accuracy in CV-E, while SINN obtained a higher genetic accuracy in CV-GE.

### Experiment 3: interpretation of feature importance and interactions

To validate the biological interpretability of the SINN model, we visualize feature importance and G×E interaction strength across the test set of the G2F2022 dataset in Fig. 5, with G×E interactions summed across SNPs per EC (b) and summed across ECs per SNP (d, e). Clay content, wind speed and organic matter content were assigned highest importance out of environmental features by SINN. The environmental features with strongest GxE were organic matter content, wind speed and magnesium saturation. Overall, soil features had both a higher feature importance and stronger interaction with SNPs.

**Fig. 5.**
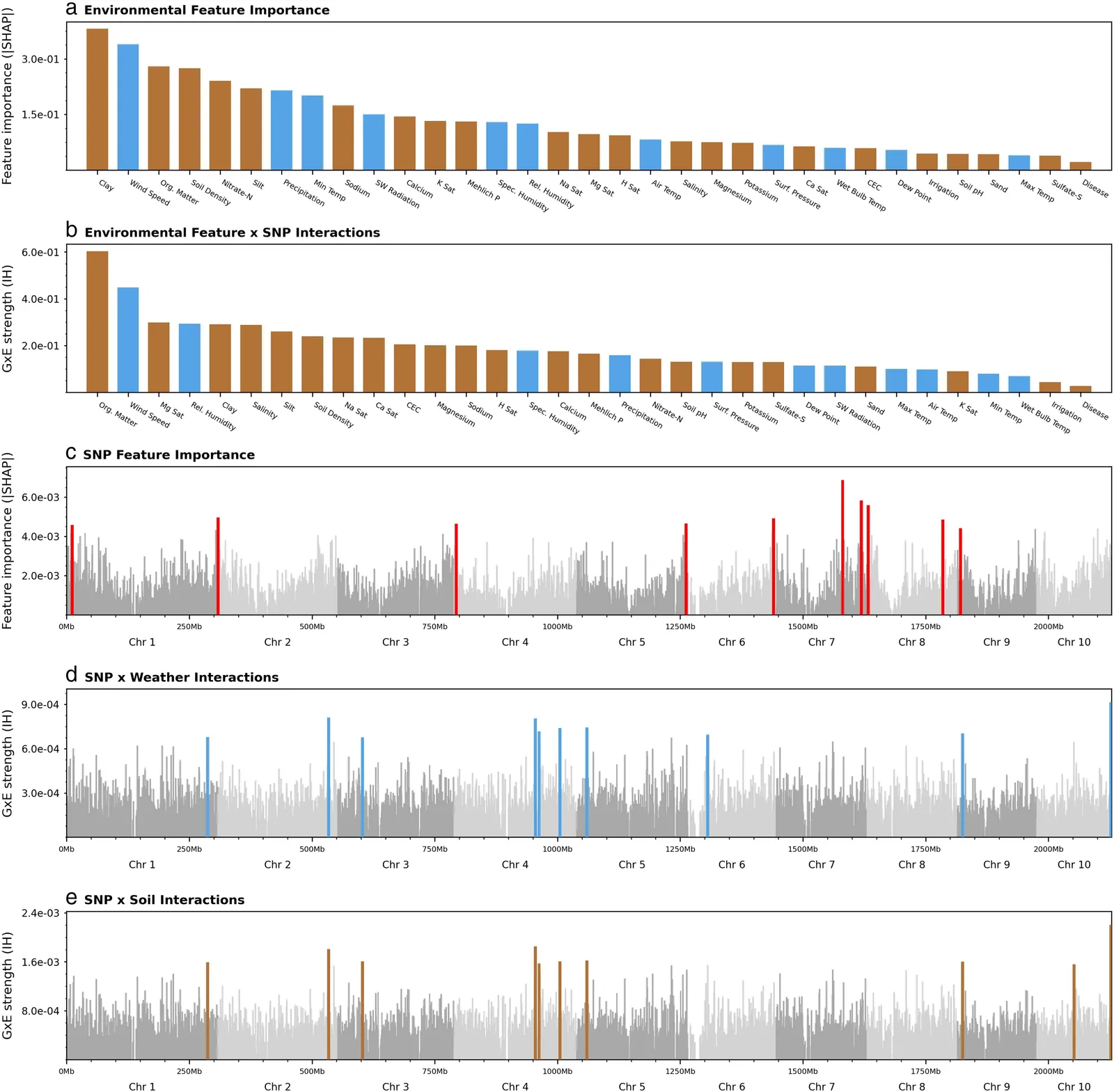
Feature importance and GxE interaction strength of SINN across G2F2022 test set. Feature importance of environmental covariates a) and SNP markers c) is quantified through SHAP values (Lundberg and Lee 2017). Interaction strength between each environmental covariate and all SNPs b) and each SNP and all weather features d) and soil features e) are quantified through Integrated Hessians (IH) (Janizek et al. 2021). In a) and b) weather features are shown in blue, and soil and management features are shown in brown. In c–e), top 10 SNPs with highest SHAP / IH values are highlighted, with red showing SNPs with highest overall importance, blue showing SNPs with strongest SNP x weather interactions and brown showing SNPs with strongest SNP x soil interactions.

In Fig. 5c–e feature importance and GxE interaction strength of individual SNPs is shown. For both feature importance and interaction strength, no clear cutoff point was identifiable. As such, the top 10 SNPs for overall importance, interaction with weather, and interaction with soil were considered relevant for further analysis. Near the end of chromosome 7 the 3 SNPs were located with the highest overall importance. We identified 3 SNPs on chromosome 4 with high interaction for both soil and weather features. Overall profile of SNP showed a weak similarity to both SNP by soil and SNP by weather interaction, while top SNPs showed no overlap. This suggests that the genetic control of yield potential is distinct from the genetic control of environmental stability in this population. Interaction with soil and weather showed a near-identical pattern, both in overall profile and top SNPs. This suggests that the identified SNPs for GxE do not confer tolerance to a specific environmental stressor, but modulate overall environmental sensitivity.

The high importance and interaction scores for wind speed, soil nutrients and soil physical properties suggest root and stem lodging as potential mechanisms underlying environmental variation and GxE. Further evidence is provided by the training data records: more than 10% of plants demonstrated root or stem lodging in 18% of training plots. Importance and interaction scores were relatively evenly distributed across ECs and SNPs, suggesting no single cause is sufficient for explaining predicted genetic variation, environmental variation or GxE. We provide an overview of SNPs identified by SINN, closest genes, and their functional annotation in Table A3 in Appendix B.

### Experiment 4: yield prediction in regional variety trials

We next investigate if the SINN framework is applicable beyond the G2F2022 dataset. We replicate the protocol of *Experiment 2* on the MaizeGEP dataset (D Zhang et al. 2026), which consists of maize variety trials conducted across China from 2017 to 2023. Again, we evaluate models on their ability to predict plot yields in the holdout final year. We show predictive performance of SINN, DNN and G×E-BLUP in Table 3. SINN performed best across RMSE, MAE, *r* and ${\bar{r}}_{e}$, while the baseline DNN demonstrated a slight advantage for genetic accuracy ${\bar{r}}_{g}$. G×E-BLUP was the least-performing model for each metric. Furthermore, the baseline DNN showed the least stable performance across replicates. Overall, SINN maintained strong and consistent performance on MaizeGEP.

**Table 3.** Predictive performance for prediction of maize yield in the final holdout year of the MaizeGEP dataset (D Zhang et al. 2026). Performance metrics of G×E-BLUP (Jarquín et al. 2014), DNN (Kick et al. 2023) and SINN (this work) are calculated by ensembling predictions of 6 models, each trained with 1 cross-validation fold. Scores are shown as *Average [95% Confidence Interval]*, calculated over 5 replicated ensembles. Best model per metric is shown in **bold**. Reported metrics are RMSE and MAE in normalized units, and Pearson correlation coefficient calculated across all samples (*r*), within environments (${\bar{r}}_{g}$) and within genotypes (${\bar{r}}_{e}$).

Model	RMSE ↓	MAE ↓	*r* ↑	${\bar{r}}_{g}$ ↑	${\bar{r}}_{e}$ ↑
GxE-BLUP	0.93 [0.93, 0.93]	0.72 [0.72, 0.72]	0.49 [0.49, 0.49]	0.26 [0.26, 0.26]	0.09 [0.09, 0.09]
DNN	0.74 [0.67, 0.80]	0.57 [0.49, 0.64]	0.76 [0.75, 0.77]	**0.31 [0.29, 0.32]**	0.32 [0.30, 0.34]
SINN	**0.66 [0.61, 0.71]**	**0.51 [0.46, 0.56]**	**0.79 [0.78, 0.80]**	0.30 [0.30, 0.30]	**0.33 [0.32, 0.34]**

## Discussion

### Structured optimization guides neural networks toward improved utilization of genetics

Both in our own baseline DNN as in the best DNN from the G2F2022 competition, we observed a low genetic accuracy of predicted yield values. This indicates a tendency of DNNs to focus on environmental information, and under-utilize genetic information in predicting yield. This is a recognized failure mode of multimodal deep learning models (Wu et al. 2022), where the model greedily focuses on a single modality of data. By separating the prediction of each unimodal signal, SINN avoids this problem and improves the utilization of genetic information. Surprisingly, SINN also improved the prediction of the unimodal effect of the dominant environmental modality, as shown by the improved genetic accuracy with respect to the baseline DNN. We attribute this to reduced overfitting of SINN compared to DNN. Overall, this enabled SINN to outperform all DNN-based baselines, even with a relatively simple model architecture.

The statistical decomposition of yield into yield components enables the design of targeted neural network components that match model complexity and regularization to the information content of each yield component. Without this targeted design the neural network components tended to overfit on both genetic and environmental information, as was shown by the high training accuracies and low validation accuracies of the baseline DNN components. With sufficient regularization, the nonlinear neural network components demonstrated an advantage over linear BLUP-based models for predicting holdout genetic and environmental effects. Surprisingly, this improvement was smallest for interaction prediction, which has both the highest number of samples as well as the expectation of being highly nonlinear. Across our interaction prediction experiments, we noticed that neural networks had a strong tendency to overfit and were limited to a very simple interaction component to successfully generalize to new environments. We theorize that adding model complexity to any component downstream of the environmental information can push the model toward overfitting on the training environments, and that this occurs at lower interaction component complexity than overfitting on the training genotypes.

However, it is important to note that the decomposition of yield into components has 2 effects on the targets: it reduces noise in the components, and also introduces systematic errors from the statistical model assumptions which propagate to downstream functions. The first was observed by comparing the relative performance of BLUP-based models and baseline DNN-based models on predicting the differences between environments. On a per-component basis, E-BLUP outperforms the DNN environment encoder in predicting ${\hat{E}}_{j}$, while DNN outperforms G×E-BLUP in predicting differences between environments when trained directly on yield. Noise has a regularizing effect on DNN optimization, so DNN model components have a stronger tendency to overfit when this noise is removed. While the regularization of the DNN model was tuned through weight decay and learning rate, this was insufficient to avoid overfitting without modifying the network architecture. However, with full tuning of regularization and model complexity, this separation becomes beneficial, as it enabled SINNs to utilize genetic information more effectively than end-to-end optimized DNNs.

### Understanding the challenges of deep learning-based interaction prediction

A commonly stated aim for applying deep learning based methods to genomic prediction in new environments is to predict nonlinear genotype-by-environment interactions. However, this aim cannot be validated based current evaluation protocols, which rely on aggregate accuracy metrics evaluated across cross-validation scenarios.

SINN enables us to measure interaction prediction performance in isolation, and thus allowed us to directly test this aim for the first time. Surprisingly, we found that the baseline DNN from Kick et al. (2023) failed to predict any genotype-by-environment interactions for holdout environments, when interactions were isolated from the genetic and environmental main effects. When evaluated directly on yield, the additive structure of BLUP-based and SINN-based methods enable quantification of the contribution of predicted interactions. However, current evaluation protocols do not allow us to evaluate whether the DNN predicts any interactions in this context. For method development, a failure to measure progress means a failure to improve. SINN allows us to measure interaction prediction performance in isolation, and thus enables the targeted improvement of DNNs for interaction prediction within the framework. However, it does not enable the measurement of interaction prediction performance for models fitted end-to-end on yield. New metrics are needed for this purpose. As a potential diagnostic tool we identify the empirical multimodally-additive function projection (EMAP) by Hessel and Lee (2020), which measures black box function performance without predicted interactions.

### Prediction of environment-specific ranking is limited by number of independent environments

In Experiment 1, we compared interaction prediction performance of models across multiple scenarios and found that all models show a large decrease in performance when predicting in new environments. We also note that a low-complexity neural network interaction component was needed to limit overfitting on environments, even though the models were trained on over 100,000 interactions. This mirrored the results of predicting ${\hat{G}}_{i}$ and ${\hat{E}}_{j}$, as prediction performance for ${\hat{E}}_{j}$ was considerably lower than for ${\hat{G}}_{j}$, and a low-complexity neural network component was needed to avoid overfitting on ${\hat{E}}_{j}$. This comes as no surprise for ${\hat{E}}_{j}$, as only 217 unique environments were available in the training set, and DNNs typically need 1,000s to 10,000s of independent samples to improve over other methods.

Surprisingly, a similar overfitting pattern was observed for the 100,000 interaction prediction samples. A potential explanation is that the interactions are not independent samples, but repeated realizations of the same 217 environments, which encourages overfitting on environmental information. This suggests that the main limitation to prediction of environment-specific genetic rankings in new environments is the limited number of unique environments, not the number of unique genotypes. This is consistent with findings of Washburn et al. (2021) that yield prediction performance of DNNs could be improved by integrating a large number of unique environments, even when these environments had low-quality yield labels. Additionally, this suggests that DNN-based approaches for predicting genetic rankings in new environments might favor recently proposed sparse MET designs (Jarquin et al. 2020; Crespo-Herrera et al. 2021), where fewer genotypes are grown in each environment in favor of more unique realizations of environments.

### SINN can utilize advanced statistical models for multi-environment trials

Error propagation from the statistical decomposition is an unwanted by-effect of SINN. Any errors in the decomposition of $y_{s}$ into ${\hat{G}}_{i}$, ${\hat{E}}_{j}$ and ${\hat{GE}}_{ijk}$ propagate through the training of ${\hat{\;f}}_{g}$, ${\hat{\;f}}_{e}$ and ${\hat{\;f}}_{ge}$ to downstream predictive ability. Addressing this error propagation completely would require integration between the statistical decomposition and the end-to-end optimization of DNNs, which we leave for further work. As a more practical approach, we propose to improve the decomposition of yield into ${\hat{G}}_{i}$, ${\hat{E}}_{j}$ and ${\hat{GE}}_{ijk}$ with statistical models for estimating genotype-environment interactions. In this work, we deliberately pursue a minimal implementation of SINN and thus utilize simple DNN components, and a minimally complex decomposition. However, as we note in section *Introducing SINN*, this naive decomposition obtains biased estimates of E[y∣i] and E[y∣j]. Fitting $G_{i}$ and $E_{j}$ as random effects could improve these estimates, as the MET in the G2F2022 dataset is imbalanced and incomplete. Furthermore, the current decomposition does not separate genotype-interaction from noise. This can be realized by a wide range of modern statistical models for both genotype-environment interaction and noise, including modeling of experimental design (blocks, rows, columns), spatial trends within trials with P-splines (Velazco et al. 2017; Rodríguez-Álvarez et al. 2017), and variance-covariance structures of genotype-environment interactions with modern factor-analytic models (Piepho and Williams 2024).

### Broader applicability

*Experiment 4* highlights broader applicability of SINN. On an independent, secondary MET dataset in a different geographic context (China), different breeding context (national variety trials) and MET dimensions (10x fewer genotypes, 10x more trials) SINN demonstrated superior performance compared to baseline statistical and deep learning-based methods. Predictive performance in national variety trials serves as strong indicator of generalizability, as these trials are designed to represent the full range of complex genotype-by-environment interactions encountered in commercial agricultural production. Furthermore, we note that national variety trials form a complementary source of data to breeding trials, as they naturally contain a large number of unique environments. We consider integration and transfer learning between these 2 types of datasets a promising and broadly applicable direction for the prediction of environment-specific rankings.

### Further potential of multimodal deep learning

We further emphasize that the sub-components of current MMDL-based approaches remain underoptimized for GEP, which suggests substantial potential for further gains in predictive performance. In this work, we demonstrate that the performance of current MMDL methods for GEP can be greatly improved by modifying only the *training algorithm* to target *imbalanced multimodal learning*. Imbalanced multimodal learning is an active area of research (Fan et al. 2023; Y Wei and Hu 2024; Y Wei et al. 2024; X Zhang et al. 2024; S Wei et al. 2025), and new insights into the problem and targeted solutions are increasingly available. Unfortunately, current approaches are specifically tailored to classification tasks in observational data. We expect adaptation of these approaches to regression tasks and structured data to yield further performance gains.

Additionally, MMDL models are built of architectural modules that can be independently improved. In this work, we demonstrated the SINN framework with a minimal MMDL network without specialized architectural modules. As such, we expect replacement of each architecture module (genomic encoder, environment encoder, interaction module) to improve prediction performance. For the genomic encoder, a wide range of specialized architectures has recently been proposed (Ma et al. 2018; Liu et al. 2019; K Wang et al. 2023; H Wang et al. 2025). For the environment encoder, we consider specialized architectures proposed in yield forecasting (Khaki et al. 2020; Jácome Galarza et al. 2025). For the interaction encoder, specialized fusion layers (Cheng et al. 2016; H Guo et al. 2017; R Wang et al. 2021; Mao et al. 2023) are expected to further improve performance.

### Future work

Our results on 2 datasets show that SINN improves over current methods in predicting the next cycle of large-scale multi-environment trials. Results across diverse crops, regions and traits are expected to build further evidence of external validity of the method in the future. We expect SINN to generalize to other settings, as SINN does not rely on any crop, region, or trait-specific knowledge, and the 2 presented case studies contained many challenging aspects in terms of genetics (2 diverse populations of maize hybrids), environmental conditions (US- and China-wide spatial scopes), predicted trait (yield) and data quality (large residual variation).

More benchmark datasets for the development of GEP models are necessary. Current datasets are either not open (Khaki and Wang 2019; Ansarifar et al. 2020), contain too few trials (Montesinos-López et al. 2023), are missing marker information (Ansarifar et al. 2020; Shook et al. 2021) or are missing environmental features (Montesinos-López et al. 2023). As the current generation of models for GEP highlights the potential for large-scale prediction of GxE in multi-environment trials, we are optimistic that more suitable datasets will become available for research in the future.

## Conclusion

We demonstrate the Structured Interaction Neural Network (SINN), a new framework for the optimization of deep neural networks (DNNs) for GEP. We show that SINN improves genomic prediction of maize yield in future environments when compared to a kernel-based statistical model, a specialized DNN, and the best models of an open benchmark dataset. Furthermore, we demonstrate consistent improvements across 2 large-scale multi-environment trials spanning both the United States and China. SINN reduces overfitting and improves prediction of genetic effects, environmental effects and genotype-by-environment interactions when compared to DNN models, and improves prediction of environmental effects when compared to kernel-based statistical models. Our findings further indicate that prediction of environment-specific rankings is mainly limited by generalization to new environments, rather than generalization to new genotypes. Surprisingly, we find that the baseline DNN had the lowest performance and failed to predict any genotype-environment interactions in new environments, when interactions are predicted in isolation.

Our SINN framework structures further MMDL research, as it decomposes both the task and model into components that can be improved independently. Additionally, it opens the door to further integration of advanced statistical decomposition methods with new DNN approaches. By bridging statistical genetics and deep learning, SINN provides a new approach to accurate, modular and scalable prediction of genotype-environment interactions.

## Data Availability

All data used in this study is freely available. The Genomes to Fields 2022 Maize Genotype by Environment Prediction Competition is available on CyVerse under https://pubmed.ncbi.nlm.nih.gov/37461058/. The MaizeGEP dataset is available at http://user.ebreed.cn:9992/scb/. Upon publication, the code and scripts used in this study will be made publicly available in the following GitHub repository: https://github.com/wur-ai/sinn.

## Appendix A: Experimental settings and hyperparameter selection

### MaizeGEP

For the MaizeGEP dataset, we applied six-fold cross-validation to models and SINN model components. We follow the same definition of CV-E used in *Experiment 1*. We use a simplified definition for CV-G: we define each validation set as the genotypes newly introduced in 1 year, matching the year of the corresponding validation set in CV-E. This maximizes the overlap in interactions between CV-G and CV-E. Finally, for interaction prediction, we do not define a separate CV-X, as this is not needed for the conducted experiments.

To replicate SINN on the MaizeGEP dataset in *Experiment 4* with minimal manual experimentation, we made some adjustments to our hyperparameter selection pipeline to improve the stability of model selection. Firstly, to reduce overfitting, we introduced cross-validation based early stopping, where we record the optimum number of training epochs across all considered cross-validation folds for each setting as additional hyperparameter. Second, to counteract per-epoch noise in evaluation, we smoothed the selection metric using an exponential moving average (EMA) with with smoothing parameter α=0.2. This pipeline was applied equally to both SINN and the baseline DNN. Furthermore, we adjusted the hyperparameter ranges and number of replicates of genotype and environment encoders to the 10x smaller number of genotypes and 10x larger number of environments. Each hyperparameter setting of the genetic encoder model was evaluated across all 6 folds, and with 2 replicate seeds per fold. Conversely, no replicate seeds were needed for the environment encoder. Exact replicates, hyperparameter ranges and final selected hyperparameters for MaizeGEP are shown in Table A2.

**Table A1.** Settings and search spaces for hyperparameter selection in the G2F2022 dataset.

Model	Target	#	Layers	Nodes per layer	Learning rate	Weight decay
G-SINN	${\hat{G}}_{i}$	125		{64, 96, 128, 192, 256}	{1e-4, 3e-4, 1e-3, 3e-3, 1e-2}	{1e-4, 3e-4, 1e-3, 3e-3, 1e-2}
E-SINN	${\hat{E}}_{j}$	200×5	{ 3, 4, 5 }	{8, 16, 32, 48, 64}	{1e-5, 3e-5, 1e-4, 3e-4, 1e-3}	{1e-5, 5e-5, 1e-4, 3e-4, 1e-3}
G×E-SINN	${\hat{GE}}_{ijk}$	125		{8, 16, 32, 64, 128 }	{1e-4, 3e-4, 1e-3, 3e-3, 1e-2}	{1e-4, 3e-4, 1e-3, 3e-3, 1e-2}
DNN (G encoder)	${\hat{G}}_{i}$	25			{1e-4, 3e-4, 1e-3, 3e-3, 1e-2}	{1e-4, 3e-4, 1e-3, 3e-3, 1e-2}
DNN (E encoder)	${\hat{E}}_{j}$	25×5			{1e-5, 3e-5, 1e-4, 3e-4, 1e-3}	{1e-5, 5e-5, 1e-4, 3e-4, 1e-3}
DNN	${\hat{GE}}_{ijk}$	25			{1e-4, 3e-4, 1e-3, 3e-3, 1e-2}	{1e-4, 3e-4, 1e-3, 3e-3, 1e-2}
DNN	$y_{s}$	25			{1e-4, 3e-4, 1e-3, 3e-3, 1e-2}	{1e-4, 3e-4, 1e-3, 3e-3, 1e-2}

**Table A2.** Settings, search spaces and final selected hyperparameters for hyperparameter selection in the MaizeGEP dataset, with selected hyperparameters in **bold**. Optimal number of training epochs selected through early stopping is also shown in **bold**.

Dataset	MaizeGEP			
Model	G-SINN	E-SINN	G×E-SINN	DNN
**Target**	${\hat{G}}_{i}$	${\hat{E}}_{j}$	${\hat{GE}}_{ijk}$	$y_{s}$
**Settings**	125	200	125	25
**Seeds**	2	1	1	1
**Folds**	6	1	1	1
**Layers**	{**2**, 3, 4, 5}	{3, 4, **5**}	–	–
**Nodes per Layer**	{16, 32, 48, **64**}	{32, 64, 96, **128**}	{8, 16, **32**, 64, 128}	–
**Learning rate**	{1e-4, **3e-4**, 1e-3, 3e-3}	{1e-5, 3e-5, **1e-4**, 3e-4, 1e-3}	{**1e-4**, 3e-4, 1e-3, 3e-3, 1e-2}	{1e-4, **3e-4**, 1e-3, 3e-3, 1e-2}
**Weight decay**	{1e-4, 3e-4, 1e-3, **3e-3**}	{1e-5, 3e-5, 1e-4, 3e-4, **1e-3**}	{1e-4, 3e-4, 1e-3, **3e-3**, 1e-2}	{1e-4, **3e-4**, 1e-3, 3e-3, 1e-2}
**Batch size**	{16, 32, **48**, 64}	**128**	**256**	**256**
**Epochs**	250 (**171**)	250 (**59**)	250 (**146**)	150 (**43**)
**Averaging days for weather features**	-	{1, 7, 30, **244**}	-	**1**

## Appendix B: SNP-gene associations

**Table A3.** Identified genes within a 5,000 bp window to top 10 SNPs for overall importance (G), interaction with weather (GxW) and interaction with soil (GxS). Functional annotations were retrieved programmatically from the UniProtKB database (Bateman et al. 2025).

SNP ID	G	GxW	GxS	Candidate Gene	Distance (bp)	Annotation
Chr1_11573869	✓			Zm00001eb004190	36	cell cycle regulation
Chr1_287395927		✓	✓	Zm00001eb058550	0	pollen tube guidance
Chr2_107829	✓			Zm00001eb065570	115	exocytosis
Chr2_225461006		✓	✓	Zm00001eb112080	0	cytokinin signaling
Chr4_166120868		✓	✓	Zm00001eb188580	0	GTP-binding protein
Chr4_173620257		✓	✓	Zm00001eb190540	38	zinc homeostasis
Chr5_20983785		✓	✓	Zm00001eb219710	1,633	triterpenoid biosynthesis
Chr5_222837505	✓			Zm00001eb258240	0	BHLH protein
Chr6_40844033		✓		Zm00001eb266050	0	rRNA processing
Chr7_173083062	✓			Zm00001eb326950	214	system development
Chr8_1829248	✓			Zm00001eb332580	0	DNA-binding protein
Chr8_153859814	✓			Zm00001eb360010	0	uncharacterized
Chr9_7799385	✓			Zm00001eb372830	90	mitochondrial heat shock protein
Chr9_11949333		✓	✓	Zm00001eb373840	506	uncharacterized
Chr10_150914480		✓	✓	Zm00001eb434080	0	brassinosteroid signaling

## References

[iyag204-B1] Ansarifar J, Akhavizadegan F, Wang L. 2020. Performance prediction of crosses in plant breeding through genotype by environment interactions. Sci Rep. 10:11533. 10.1038/s41598-020-68343-1. PMC7359316 32661366

[iyag204-B2] Ba JL, Kiros JR, Hinton GE. 2016. Layer normalization, arXiv preprint arXiv:1607.06450.

[iyag204-B3] Bateman A et al 2025. Uniprot: the universal protein knowledgebase in 2025. Nucleic Acids Res. 53:609–617. 10.1093/nar/gkae1010. PMC11701636 39552041

[iyag204-B4] Cadene R, Dancette C, Ben-younes H, Cord M, Parikh D. 2019. Rubi: reducing unimodal biases for visual question answering. In: Advances in Neural Information Processing Systems; Vol. 32.

[iyag204-B5] Chapman SC, Chakraborty S, Dreccer MF, Howden SM. 2012. Plant adaptation to climate change–opportunities and priorities in breeding. Crop Pasture Sci. 63:251–268. 10.1071/CP11303.

[iyag204-B6] Cheng HT et al 2016. Wide & deep learning for recommender systems. In: Proceedings of the 1st Workshop on Deep Learning for Recommender Systems; p. 7–10.

[iyag204-B7] Cooper M, Powell O, Gho C, Tang T, Messina C. 2023. Extending the breeder’s equation to take aim at the target population of environments. Front Plant Sci. 14:1129591. 10.3389/fpls.2023.1129591. PMC9990092 36895882

[iyag204-B8] Cooper M, Technow F, Messina C, Gho C, Totir LR. 2016. Use of crop growth models with whole-genome prediction: application to a maize multienvironment trial. Crop Sci. 56:2141–2156. 10.2135/cropsci2015.08.0512.

[iyag204-B9] Crespo-Herrera L et al 2021. Genome-enabled prediction for sparse testing in multi-environmental wheat trials. Plant Genome. 14:e20151. 10.1002/tpg2.20151. PMC12806883 34510790

[iyag204-B10] Du C et al 2023. On uni-modal feature learning in supervised multi-modal learning. In: International Conference on Machine Learning; PMLR. p. 8632–8656.

[iyag204-B11] Fan Y, Xu W, Wang H, Wang J, Guo S. 2023. Pmr: Prototypical modal rebalance for multimodal learning. In: Proceedings of the IEEE/CVF Conference on Computer Vision and Pattern Recognition; p. 20029–20038.

[iyag204-B12] Fernandes IK, Vieira CC, Dias KO, Fernandes SB. 2024. Using machine learning to combine genetic and environmental data for maize grain yield predictions across multi-environment trials. Theor Appl Genet. 137:189. 10.1007/s00122-024-04687-w. PMC11266441 39044035

[iyag204-B13] Goodfellow I, Bengio Y, Courville A. 2016. Deep Learning. Vol. 1. MIT Press Cambridge.

[iyag204-B14] Guo H, Tang R, Ye Y, Li Z, He X. 2017. Deepfm: a factorization-machine based neural network for ctr prediction. In: Proceedings of the Twenty-Sixth International Joint Conference on Artificial Intelligence. p. 1725–1731.

[iyag204-B15] Guo J et al 2020. Multi-trait genomic prediction of yield-related traits in us soft wheat under variable water regimes. Genes (Basel). 11:1270. 10.3390/genes11111270. PMC7716228 33126620

[iyag204-B16] He K et al 2025. Leveraging automated machine learning for environmental data-driven genetic analysis and genomic prediction in maize hybrids. Adv Sci. 12:2412423. 10.1002/advs.202412423. PMC12061318 40047344

[iyag204-B17] Heslot N, Akdemir D, Sorrells ME, Jannink JL. 2014. Integrating environmental covariates and crop modeling into the genomic selection framework to predict genotype by environment interactions. Theor Appl Genet. 127:463–480. 10.1007/s00122-013-2231-5. 24264761

[iyag204-B18] Hessel J, Lee L. 2020. Does my multimodal model learn cross-modal interactions? It’s harder to tell than you might think! In: Proceedings of the 2020 Conference on Empirical Methods in Natural Language Processing (EMNLP); p. 861–877.

[iyag204-B19] Hestness J et al 2017. Deep learning scaling is predictable, empirically, arXiv preprint arXiv:1712.00409.

[iyag204-B20] Holland JB, Piepho HP. 2024. Don’t blup twice. G3 (Bethesda). 14:jkae250. 10.1093/g3journal/jkae250. PMC11631391 39558791

[iyag204-B21] Hu H, Rincent R, Runcie DE. 2025. Megalmm improves genomic predictions in new environments using environmental covariates. Genetics. 229:1–41. 10.1093/genetics/iyae171. PMC11708919 39471330

[iyag204-B22] Huang Y, Lin J, Zhou C, Yang H, Huang L. 2022. Modality competition: what makes joint training of multi-modal network fail in deep learning? (provably). In: International Conference on Machine Learning; PMLR. p. 9226–9259.

[iyag204-B23] Jácome Galarza L, Realpe M, Viñán-Ludeña MS, Calderón MF, Jaramillo S. 2025. Agritransformer: a transformer-based model with attention mechanisms for enhanced multimodal crop yield prediction. Electronics (Basel). 14:2466. 10.3390/electronics14122466.

[iyag204-B24] Janizek JD, Sturmfels P, Lee SI. 2021. Explaining explanations: axiomatic feature interactions for deep networks. J Mach Learn Res. 22:1–54.

[iyag204-B25] Jarquín D et al 2014. A reaction norm model for genomic selection using high-dimensional genomic and environmental data. Theor Appl Genet. 127:595–607. 10.1007/s00122-013-2243-1. PMC3931944 24337101

[iyag204-B26] Jarquin D et al 2020. Genomic prediction enhanced sparse testing for multi-environment trials. G3 (Bethesda). 10:2725–2739. 10.1534/g3.120.401349. PMC7407457 32527748

[iyag204-B27] Jubair S, Domaratzki M. 2023. Crop genomic selection with deep learning and environmental data: a survey. Front Artif Intell. 5:1040295. 10.3389/frai.2022.1040295. PMC9871498 36703955

[iyag204-B28] Jubair S, Tremblay-Savard O, Domaratzki M. 2023. Gxenet: novel fully connected neural network based approaches to incorporate gxe for predicting wheat yield. Artif Intell Agric. 8:60–76. 10.1016/j.aiia.2023.05.001.

[iyag204-B29] Jullien A et al 2011. Characterization of the interactions between architecture and source–sink relationships in winter oilseed rape (brassica napus) using the greenlab model. Ann Bot. 107:765–779. 10.1093/aob/mcq205. PMC3077979 20980324

[iyag204-B30] Kang Y, Khan S, Ma X. 2009. Climate change impacts on crop yield, crop water productivity and food security–a review. Prog Nat Sci. 19:1665–1674. 10.1016/j.pnsc.2009.08.001.

[iyag204-B31] Khaki S, Wang L. 2019. Crop yield prediction using deep neural networks. Front Plant Sci. 10:621. 10.3389/fpls.2019.00621. PMC6540942 31191564

[iyag204-B32] Khaki S, Wang L, Archontoulis SV. 2020. A cnn-rnn framework for crop yield prediction. Front Plant Sci. 10:1750. 10.3389/fpls.2019.01750. PMC6993602 32038699

[iyag204-B33] Kick DR et al 2023. Yield prediction through integration of genetic, environment, and management data through deep learning. G3 (Bethesda). 13:jkad006. 10.1093/g3journal/jkad006. PMC10085787 36625555

[iyag204-B34] Lawrence-Dill CJ, Schnable PS, Springer NM. 2019. Idea factory: the maize genomes to fields initiative. Crop Sci. 59:1406–1410. 10.2135/cropsci2019.02.0071.

[iyag204-B35] Lima DC et al 2023. Genomes to fields 2022 maize genotype by environment prediction competition. BMC Res Notes. 16:148. 10.1186/s13104-023-06421-z. PMC10353085 37461058

[iyag204-B36] Liu Y et al 2019. Phenotype prediction and genome-wide association study using deep convolutional neural network of soybean. Front Genet. 10:1091. 10.3389/fgene.2019.01091. PMC6883005 31824557

[iyag204-B37] Loshchilov I, Hutter F. 2019. Decoupled weight decay regularization. In: International Conference on Learning Representations.

[iyag204-B38] Lundberg SM, Lee SI. 2017. A unified approach to interpreting model predictions. In: Advances in Neural Information Processing Systems; Vol. 30.

[iyag204-B39] Ma W et al 2018. A deep convolutional neural network approach for predicting phenotypes from genotypes. Planta. 248:1307–1318. 10.1007/s00425-018-2976-9. 30101399

[iyag204-B40] Makino T, Wang Y, Geras KJ, Cho K. 2023. Detecting incidental correlation in multimodal learning via latent variable modeling. Transact Mach Learn Res.

[iyag204-B41] Måløy H, Windju S, Bergersen S, Alsheikh M, Downing KL. 2021. Multimodal performers for genomic selection and crop yield prediction. Smart Agric Technol. 1:100017. 10.1016/j.atech.2021.100017.

[iyag204-B42] Mao K et al 2023. Finalmlp: an enhanced two-stream mlp model for ctr prediction. In: Proceedings of the AAAI Conference on Artificial Intelligence; Vol. 37. p. 4552–4560.

[iyag204-B43] Meuwissen TH, Hayes BJ, Goddard M. 2001. Prediction of total genetic value using genome-wide dense marker maps. Genetics. 157:1819–1829. 10.1093/genetics/157.4.1819. PMC1461589 11290733

[iyag204-B44] Montesinos-López A et al 2023. Multimodal deep learning methods enhance genomic prediction of wheat breeding. G3 (Bethesda). 13:jkad045. 10.1093/g3journal/jkad045. PMC10151399 36869747

[iyag204-B45] Montesinos-López A, Montesinos-López OA, Gianola D, Crossa J, Hernández-Suárez CM. 2018. Multi-environment genomic prediction of plant traits using deep learners with dense architecture. G3 (Bethesda). 8:3813–3828. 10.1534/g3.118.200740. PMC6288841 30291107

[iyag204-B46] Montesinos-López OA et al 2019. Multi-trait, multi-environment genomic prediction of durum wheat with genomic best linear unbiased predictor and deep learning methods. Front Plant Sci. 10:1311. 10.3389/fpls.2019.01311. PMC6856087 31787990

[iyag204-B47] Montesinos-López OA et al 2024. A review of multimodal deep learning methods for genomic-enabled prediction in plant breeding. Genetics. 228:iyae161. 10.1093/genetics/iyae161. PMC11631469 39499217

[iyag204-B48] Nair V, Hinton GE. 2010. Rectified linear units improve restricted boltzmann machines. In: International Conference on Machine Learning; p. 807–814.

[iyag204-B49] Ngiam J et al 2011. Multimodal deep learning. In: International Conference on Machine Learning; Vol. 11. p. 689–696.

[iyag204-B50] Peng X, Wei Y, Deng A, Wang D, Hu D. 2022. Balanced multimodal learning via on-the-fly gradient modulation. In: Proceedings of the IEEE/CVF Conference on Computer Vision and Pattern Recognition; p. 8238–8247.

[iyag204-B51] Piepho HP, Williams E. 2024. Factor-analytic variance–covariance structures for prediction into a target population of environments. Biom J. 66:e202400008. 10.1002/bimj.202400008. 39049627

[iyag204-B52] Rincent R et al 2019. Using crop growth model stress covariates and ammi decomposition to better predict genotype-by-environment interactions. Theor Appl Genet. 132:3399–3411. 10.1007/s00122-019-03432-y. 31562567

[iyag204-B53] Rodríguez-Álvarez MX, Boer MP, van Eeuwijk FA, Eilers PH. 2017. Correcting for spatial heterogeneity in plant breeding experiments with p-splines. Spat Stat. 23:52–71. 10.1016/j.spasta.2017.10.003.

[iyag204-B54] Rosenblatt F . 1958. The perceptron: a probabilistic model for information storage and organization in the brain. Psychol Rev. 65:386–408. 10.1037/h0042519. 13602029

[iyag204-B55] Sandhu K, Patil SS, Pumphrey M, Carter A. 2021. Multitrait machine-and deep-learning models for genomic selection using spectral information in a wheat breeding program. Plant Genome. 14:e20119. 10.1002/tpg2.20119. 34482627

[iyag204-B56] Shook J et al 2021. Crop yield prediction integrating genotype and weather variables using deep learning. PLoS One. 16:e0252402. 10.1371/journal.pone.0252402. PMC8211294 34138872

[iyag204-B57] Srivastava N, Hinton G, Krizhevsky A, Sutskever I, Salakhutdinov R. 2014. Dropout: a simple way to prevent neural networks from overfitting. J Mach Learn Res. 15:1929–1958.

[iyag204-B58] Technow F, Messina CD, Totir LR, Cooper M. 2015. Integrating crop growth models with whole genome prediction through approximate bayesian computation. PLoS One. 10:e0130855. 10.1371/journal.pone.0130855. PMC4488317 26121133

[iyag204-B59] Togninalli M et al 2023. Multi-modal deep learning improves grain yield prediction in wheat breeding by fusing genomics and phenomics. Bioinformatics. 39:btad336. 10.1093/bioinformatics/btad336. PMC10246581 37220903

[iyag204-B60] Velazco JG et al 2017. Modelling spatial trends in sorghum breeding field trials using a two-dimensional p-spline mixed model. Theor Appl Genet. 130:1375–1392. 10.1007/s00122-017-2894-4. PMC5487705 28374049

[iyag204-B61] Wang H et al 2025. Cropformer: an interpretable deep learning framework for crop genomic prediction. Plant Commun. 6:101223. 10.1016/j.xplc.2024.101223. PMC11956090 39690739

[iyag204-B62] Wang K et al 2023. Dnngp, a deep neural network-based method for genomic prediction using multi-omics data in plants. Mol Plant. 16:279–293. 10.1016/j.molp.2022.11.004. 36366781

[iyag204-B63] Wang R et al 2021. Dcn v2: improved deep & cross network and practical lessons for web-scale learning to rank systems. In: Proceedings of the Web Conference 2021; p. 1785–1797.

[iyag204-B64] Wang W, Tran D, Feiszli M. 2020. What makes training multi-modal classification networks hard? In: Proceedings of the IEEE/CVF Conference on Computer Vision and Pattern Recognition; p. 12695–12705.

[iyag204-B65] Washburn JD et al 2021. Predicting phenotypes from genetic, environment, management, and historical data using cnns. Theor Appl Genet. 134:3997–4011. 10.1007/s00122-021-03943-7. 34448888

[iyag204-B66] Washburn JD et al 2025. Global genotype by environment prediction competition reveals that diverse modeling strategies can deliver satisfactory maize yield estimates. Genetics. 229:iyae195. 10.1093/genetics/iyae195. PMC12054733 39576009

[iyag204-B67] Wei S, Luo C, Luo Y. 2025. Improving multimodal learning via imbalanced learning. In: Proceedings of the IEEE/CVF International Conference on Computer Vision; p. 2250–2259.

[iyag204-B68] Wei Y, Hu D. 2024. Mmpareto: boosting multimodal learning with innocent unimodal assistance. In: International Conference on Machine Learning; PMLR. p. 52559–52572.

[iyag204-B69] Wei Y, Li S, Feng R, Hu D. 2024. Diagnosing and re-learning for balanced multimodal learning. In: European Conference on Computer Vision; Springer. p. 71–86.

[iyag204-B70] Westhues CC et al 2021. Prediction of maize phenotypic traits with genomic and environmental predictors using gradient boosting frameworks. Front Plant Sci. 12:699589. 10.3389/fpls.2021.699589. PMC8647909 34880880

[iyag204-B71] Wörtwein T, Sheeber L, Allen N, Cohn J, Morency LP. 2022. Beyond additive fusion: learning non-additive multimodal interactions. In: Findings of the Association for Computational Linguistics: EMNLP 2022; p. 4681–4696.

[iyag204-B72] Wu N, Jastrzebski S, Cho K, Geras KJ. 2022. Characterizing and overcoming the greedy nature of learning in multi-modal deep neural networks. In: International Conference on Machine Learning; PMLR. p. 24043–24055.

[iyag204-B73] Xu Y . 2016. Envirotyping for deciphering environmental impacts on crop plants. Theor Appl Genet. 129:653–673. 10.1007/s00122-016-2691-5. PMC4799247 26932121

[iyag204-B74] Yao Z et al 2025. Geformer: a genotype-environment interaction-based genomic prediction method that integrates the gating multilayer perceptron and linear attention mechanisms. Mol Plant. 18:527–549. 10.1016/j.molp.2025.01.020. 39881541

[iyag204-B75] Zhang D et al 2026. Maizegep: a maize hybrids dataset with genotype, phenotype, and envirotype to develop genomic selection models. Genomics Proteomics Bioinformatics. qzaf140. 10.1093/gpbjnl/qzaf140. 41485082

[iyag204-B76] Zhang X, Yoon J, Bansal M, Yao H. 2024. Multimodal representation learning by alternating unimodal adaptation. In: Proceedings of the IEEE/CVF conference on computer vision and pattern recognition; p. 27456–27466.

[iyag204-B77] Zhang Y, Latham PE, Saxe AM. 2024. Understanding unimodal bias in multimodal deep linear networks. In: International Conference on Machine Learning; PMLR. p. 59100–59125.

[iyag204-B78] Zhao T, Zeng J, Cheng H. 2022. Extend mixed models to multilayer neural networks for genomic prediction including intermediate omics data. Genetics. 221:iyac034. 10.1093/genetics/iyac034. PMC9071534 35212766

